# Inter-domain Communication Mechanisms in an ABC Importer: A Molecular Dynamics Study of the MalFGK_2_E Complex

**DOI:** 10.1371/journal.pcbi.1002128

**Published:** 2011-08-04

**Authors:** A. Sofia F. Oliveira, António M. Baptista, Cláudio M. Soares

**Affiliations:** Instituto de Tecnologia Química e Biológica, Universidade Nova de Lisboa, Oeiras, Portugal; University of California San Diego, United States of America

## Abstract

ATP-Binding Cassette transporters are ubiquitous membrane proteins that convert the energy from ATP-binding and hydrolysis into conformational changes of the transmembrane region to allow the translocation of substrates against their concentration gradient. Despite the large amount of structural and biochemical data available for this family, it is still not clear how the energy obtained from ATP hydrolysis in the ATPase domains is “transmitted” to the transmembrane domains. In this work, we focus our attention on the consequences of hydrolysis and inorganic phosphate exit in the maltose uptake system (MalFGK_2_E) from *Escherichia coli*. The prime goal is to identify and map the structural changes occurring during an ATP-hydrolytic cycle. For that, we use extensive molecular dynamics simulations to study three potential intermediate states (with 10 replicates each): an ATP-bound, an ADP plus inorganic phosphate-bound and an ADP-bound state. Our results show that the residues presenting major rearrangements are located in the A-loop, in the helical sub-domain, and in the “EAA motif” (especially in the “coupling helices” region). Additionally, in one of the simulations with ADP we were able to observe the opening of the NBD dimer accompanied by the dissociation of ADP from the ABC signature motif, but not from its corresponding P-loop motif. This work, together with several other MD studies, suggests a common communication mechanism both for importers and exporters, in which ATP-hydrolysis induces conformational changes in the helical sub-domain region, in turn transferred to the transmembrane domains via the “coupling helices”.

## Author Summary

ABC transporters are membrane proteins that couple ATP binding and hydrolysis with the active transport of substrates across membranes. These transporters form one of the largest families of membrane proteins and they can be found in all phyla of life. Moreover, some members of this family are involved in several genetic diseases (such as cystic fibrosis) and in multidrug resistance in bacteria, fungi and mammals. In this work, we use molecular dynamics simulations to study conformational changes due to ATP hydrolysis in an ABC transporter responsible for maltose uptake in *E. coli*. These conformational changes arising from one side of the protein (NBDs – Nucleotide Binding domains) where ATP binds, are propagated across the protein to more distant regions. Additionally, we can observe an NBD dimer interface dissociation event upon inorganic phosphate exit. These simulations together with other theoretical studies suggest that there is a general inter-domain communication mechanism common to importers and exporters.

## Introduction

The ATP-binding cassette (ABC) transporters family [1]–[3] is one of the largest class of transporters known and they are expressed ubiquitously in all kingdoms of life [1]–[3]. The members of this family play essential roles in many cellular processes, as they couple the energy gained from ATP hydrolysis [4], [5] to the transport of an enormous variety of solutes, or allocrites, (such as, inorganic ions, peptides, lipids, antibiotics, pharmacological drugs) across cellular membranes against the concentration gradient. Human ABC transporters are involved in several genetic diseases (such as bleeding disorders [6], eye [7] and liver diseases [8], and cystic fibrosis [9], [10]). There are several ABC transporters in humans that, when over expressed, confer resistance to a wide variety of chemotherapeutic drugs [11]. Additionally, several ABC transporters have also been implicated in antibiotic resistance in bacteria [11], drug resistance in fungi [11] and herbicide resistance in plants [11].

**Figure 1:**
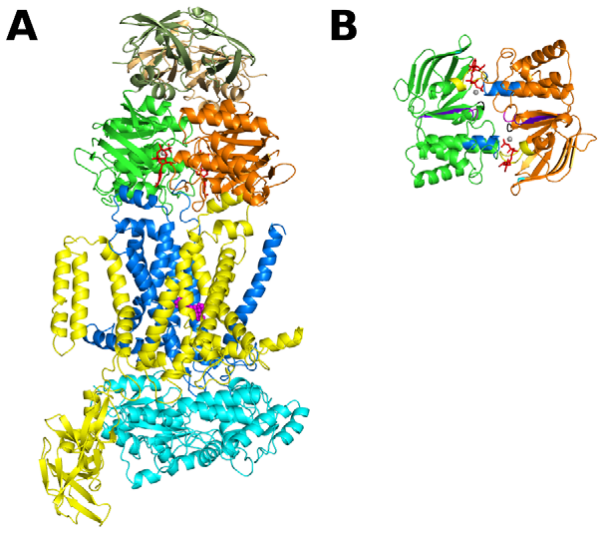
Crystallographic structure for the ATP-bound MalFGK_2_E complex (PDB code: 2R6G) [**21**]. The NBD_1_ and the NBD_2_ (named MalK_1_ and MalK_2_) are colored in green and orange respectively, while the TMD_1_ (called MalF) is yellow, the TMD_2_ (named MalG) is blue and the maltose binding protein (called MalE) is cyan. ATP is represented by red sticks, the magnesium cofactors as gray spheres and the maltose molecule is represented by magenta spheres. All figures were generated with the program PyMOL [99]. **A**- View of the MalFGK_2_E complex. In this image, the regulatory domains are colored in lighter green and orange. **B**- Rotated (90°) view of the MalK dimer. The conserved sequence family motifs are colored: Q-loop (magenta), ABC signature (blue), P-loop (yellow), A-loop (cyan), Walker-B (purple) and H-motif (black). For clarity purposes, the regulatory domains are not represented in this image.

ABC transporters comprise importers, which translocate allocrites to the cellular interior, and exporters, which do the opposite. Until now, importers have only been found in prokaryotes, whereas exporters are ubiquitously expressed in all kingdoms [12]. Independently of the transport directionality, ABC transporters are usually composed by a minimum “functional core” formed by four modules (Figure 1A): two transmembrane domains (TMDs) and two catalytic domains (NBDs). All four basic domains can be expressed as separated polypeptides or can be fused together, virtually in all possible combinations [13].

The molecular architecture of ABC transporters is nowadays well established (manly due to X-ray crystallography studies) and it is generally accepted that the TMDs recognize and provide the passageway for allocrites across the membrane, whereas the NBDs bind/hydrolyze ATP and power the transport. The low sequence conservation of the TMDs is thought to reflect the large diversity of allocrites transported, while the NBDs high sequence and structure conservation may suggest a conserved powering mechanism for all ABC transporters (both importers and exporters). The NBDs are always located in the cytoplasmic side of the membrane and are formed by two distinct sub-domains (the RecA-like and the helical sub-domains). Each sub-domain contains several characteristic sequence motifs (Figure 1B) associated with nucleotide binding and hydrolysis (e.g. the P-loop, ABC signature motif, A-loop, H-loop and Walker B motifs) [14], [15]. The two NBDs form a head-to-tail dimer (Figure 1A and 1B) with the two nucleotide molecules bound at the interface between the P-loop of one monomer and the ABC signature motif of the other [16]. Additionally to the four basic domains, ABC importers require a supplementary substrate binding protein, whose function is to capture the allocrite molecules and to deliver them to the transmembrane face of the transporter [2].

Based on the comparison of several full-length X-ray structures for both importers [17]–[24] and exporters [25]–[28], it is now clear that the structures for these two types of ABC transporters differ significantly, mainly in the transmembrane region. Nevertheless, a general transport mechanism has been hypothesized [12] based on the NBDs similarity throughout the family and in the existence of “coupling helices” in the TMDs (small α-helices oriented parallel to the membrane which directly interact with the NBDs), both in importers and exporters. In this unified mechanism, the ATP energy (whether from binding and/or hydrolysis) is converted into conformational changes, which are then transmitted from the NBDs to the TMDs, ultimately allowing active transport of the allocrites [26], [29].

The maltose/maltodextrin uptake system from *E. coli* is one of the best functionally characterized ABC transporters, importing maltooligosaccharides up to seven glucose units long [30]. This import system is formed by two different integral membrane proteins (MalF and MalG), two copies of an ABC module (MalK) and a periplasmatic binding protein (MalE) [21] (see Figure 1A). In this transporter, the ABC monomers (MalK) differ from the majority of other ABC ATPases, since they contain an extra regulatory domain (RD) in the C-terminus region, additionally to the highly conserved NBD (see Figure 1A). The RD, which is about 135 residues long, is known to interact with regulatory proteins, such as the transcriptional regulator MalT [31] or the enzyme IIA (a glucose-specific permease from the glucose-phosphotransferase system [32]–[34]). The RDs play a key role in the stabilization of the NBD dimer and, based on the X-ray structures available for the MalK dimer [21], [24], [35]–[37] (from different organisms and crystallized in several nucleotide-bound conditions), it is now possible to have a detailed view of the NBD dimer functioning mechanism and to understand the molecular basis of the increased stability of the nucleotide-free and nucleotide-bound MalK dimer. This increased stability is associated with the subunit-subunit interactions involving the RDs of both monomers [36]. In the nucleotide-free MalK dimer structure [24], [36], the NBD regions are separated (to different degrees depending whether they are in the open or semi-open state) and the dimer is solely maintained by contacts between the C-terminal RDs. Upon ATP binding [21], [36], the helical sub-domains move forward and close the NBD dimer (with the ATP molecules trapped in the binding sites).

Despite the large amount of experimental data available for the ABC transporter family (including high-resolution X-ray structures for several full-length members [17]–[28]), many fundamental questions are still waiting to be answered. In particular, how is the energy released from nucleotide hydrolysis converted into mechanical work, in order to allow unidirectional allocrite transport and how are the conformational rearrangements induced by ATP hydrolysis transmitted from the NBDs to the TMDs, allowing allocrite passage. Understanding the detailed mechanism of transport requires the knowledge of the transporter dynamics and associated conformational changes. Molecular dynamics (MD) simulation techniques (with sufficient simulation time and conformational sampling) are a good way to study the dynamic behavior of the transporters. In the last decade, several studies using MD simulations have been reported (including by ourselves [38], [39]) for several isolated ABC domains [38], [40]–[48] or for complete ABC members [39], [49]–[60], aiming, not only to study the dynamic behavior of the ABC proteins, but also the structural transitional pathways between conformations. Some of these works even used the isolated ATPase domains of the maltose transporter as a model [43], [45]. A recent work from our laboratory [39] reported ATP hydrolysis dependent conformational changes in a complete prokaryotic exporter. Besides conformational changes in the NBDs themselves, mostly in the helical sub-domains (similarly to what was observed in our studies on isolated NBDs [38], [48]), we saw evidences of conformational changes in the TMDs, especially in the zones of the extra-cellular loops and the coupling helices. Additionally, we saw, in the post-hydrolysis state only, NBD dimer dissociation.

The main objective of this work is to map the short time scale response (<50 ns) of an ABC importer to nucleotide hydrolysis and inorganic phosphate exit. For this purpose, we simulated the MalFGK_2_E complex in three intermediate states of the ATP-catalytic cycle: a pre-hydrolysis state (with two ATP molecules bound in the nucleotide binding sites), a post-hydrolysis state (with two ADPs and two inorganic phosphate species bound) and a post-IP exit state (with two ADPs bound). In order to reduce the known sampling problem in MD simulation of proteins [61], ten 50-ns replicates were performed for each system. Based on our simulations, and using the maltose uptake system as a prototype for importers, we were able to identify the ATP-dependent conformational changes, as well as the residues responsible for inter-domain communication in ABC importers. Additionally, we compared the major conformational changes observed for the maltose importer with our previous study on an ABC exporter [39], in order to infer about a potentially general communication mechanism in the ABC transporter family.

## Materials and Methods

### Starting structure

The 2.8 Å resolution crystal structure of the E159Q mutant MalFGK_2_E transporter (PDB code: 2R6G) [21] was used as the starting point for this work. In this crystal structure, the “hydrolytic” glutamate (located in the nucleotide binding site region, and in close proximity to the γ-phosphate of ATP), was mutated to a glutamine, producing a hydrolysis deficient transporter [21]. In our simulations, the mutation was reverted and the glutamine residue was substituted back to glutamate, in order to recover the wild type transporter. Additionally, in the X-ray structure, there were several missing atoms and residues, which were modeled with the program MODELLER 6v1 [62] in order to reconstruct the complete transporter structure. For this reconstruction, 10 different models were generated and the one with the lowest value for MODELLER's objective function was selected.

The protonation state of each protonatable group was determined using a combination of Poisson-Boltzmann calculations, performed with the package MEAD (version2.2.5) [63]–[65], and Metropolis Monte Carlo simulations, using the program PETIT (version1.3) [66]. For details related with the determination of the protonation state of the protonatable residues, see section 1 in Text S1.

#### MalFGK_2_E insertion into a lipid bilayer

Our reconstructed ATP-bound MalFGK_2_E complex was inserted in a pre-equilibrated dimysristoylphosphatidylcholine (DMPC) lipid bilayer (for details related with the membrane construction and equilibration as well as its characterization, see section 2 in Text S1). The MalF and MalG optimal position relative to the membrane was determined based on the hydrophobicity of the protein's residues. After MalF and MalG insertion into the membrane, all lipids within a cut-off distance of 1.2 Å from the protein atoms were rejected, as described by other authors [67]. Subsequently, the system (protein and membrane) was hydrated using a pre-equilibrated box of SPC water molecules [68] in a rectangular box. The central cavity formed by MalF and MalG was also filled with water molecules. The water molecules misplaced in the center of the bilayer (which is formed by the highly hydrophobic DMPC tails), were removed after visual inspection. In the final system, the ATP-bound MalFGK_2_E complex is embedded in a 403 DMPC bilayer and surrounded by 77018 water molecules, in a total of 268394 atoms.

#### General setup for the molecular dynamics simulations

All MD simulations were performed using the GROMACS 3.3.1 package [69], [70] and the 53A6 GROMOS96 force field [71]–[74]. The parameters for the nucleotides species (ATP, ADP and IP) were taken from our previously published work [38], whereas the parameters for the maltose molecule were obtain from the 45A4 GROMOS96 force field [75]. The parameters for DMPC lipid molecules were taken from the 53A6 GROMOS96 force-field, except for the atomic partial charges, which were the ones derived by Chiu et al. [76]. All simulations were performed at the constant temperature of 310 K. The temperature of the system was coupled using a Berendsen heat bath [77] with a coupling constant of 0.1 ps, and separate coupling of solutes (protein, nucleotides and lipids) and solvent. The chosen temperature for the simulations (310 K) is above the phase transition temperature for the DMPC lipids (Tm = 296–297 K) in order to ensure that the bilayer is in the liquid crystalline state [78]. The pressure was coupled semi-isotropically (coupling constant of 6.0 ps and compressibility of 4.5×10^−5^ bar^−1^), resulting in independent coupling of the lateral P(x+y) and perpendicular (Pz) pressures. For all simulations, the z pressure component was kept at 1 atm, and the x and y components were calculated in order to obtain a surface tension of 25 dynes/cm (which was shown to give the correct properties for the DMPC lipid using the 53A6 GROMOS force-field [79]). The SETTLE algorithm [80] was used for keeping the bond length and the angle of water molecules at their equilibrium values, and the LINCS algorithm [81] was used to keep all remaining bonds constrained. The non-bonded interactions were calculated using a twin range method [82] with short and long-range cut-offs of 8 and 14 Å, respectively. A reaction field correction for truncated electrostatic interactions [83], [84] was applied, considering a dielectric constant of 54 [85]. The time step for integrating the equations of motion was 0.002 ps and the neighbor lists were updated every 5 steps.

The ATP-bound MalFGK_2_E complex (hereafter designated **2ATP**) was first energy minimized to remove excessive strain. Initially, we performed 5000 steps of steepest descent minimization with harmonic restraints (with a force constant of 1000 kJ mol^−1^ nm^−2^) applied to all heavy atoms, followed by another 5000 steps of the same algorithm, only restraining the protein heavy atoms, ending with 5000 steps with restraints applied to the Cα atoms only. After the minimization procedure, and in order to allow proper repacking of the lipids around the protein, we performed 500 ps of MD simulation with all protein, nucleotides and cofactors atoms harmonically restrained (the force constant used was 1000 kJ mol^−1^ nm^−2^), at constant temperature and pressure. Afterwards, 250 ps of MD simulation were calculated, with position restraints (the force constant used was 750 kJ mol^−1^ nm^−2^) applied to the protein non-hydrogen atoms only. Finally, only the Cα atoms were restrained (the force constant used was 500 kJ mol^−1^ nm^−2^) for a period of 250 ps. The **2ATP** unrestrained simulations started after these 1-ns restrained simulations.

After 10 ns of unrestrained MD simulations for the **2ATP** state (see Figure S3 in Text S1, section 3), two new systems were built by transforming the original ATP nucleotides in ADP+IP (hereafter designated **2ADP.IP**) or in ADP (hereafter designated **2ADP**). The **2ATP** simulations continued for 40 ns more, accompanied now by these two new sets of correlated simulations, that were calculated up to 50 ns in length. The conversion of ATP into ADP or ADP+IP was performed in a 5 ps time interval using a “slow growth” method [86], were the ATP atoms were changed to create the new nucleotide species.

In order to reduce the sampling problems in protein simulations, ten MD simulations, 50 ns each, were calculated for each state, in a total of thirty simulations, resulting in 1.5 µs of total simulation time. All replicates were initiated with different sets of random velocities.

### Data analysis

All average structures were computed after Cα least-squares fitting to the X-ray structure, and by neglecting the first 40 ns of simulation.

The atomic positional deviations were calculated by comparing the different nucleotide bound states (**2ADP.I**P and **2ADP**) to the **2ATP** systems, within the same replicate, and averaging these differences over all replicates (as described in [38]).

The secondary structure assignment was done using the DSSP program [87]. To determine the percentage of secondary structure loss relative to the X-ray structure, the regular secondary structure classes considered were the α-helix, the β-sheet, the 3_10_ helix and the β-bridge (DSSP classification). Only the residues that remain in the same secondary structure class as in the X-ray structure are counted.

## Results/Discussion

### MalFGK_2_E structural stability

**Figure 2:**
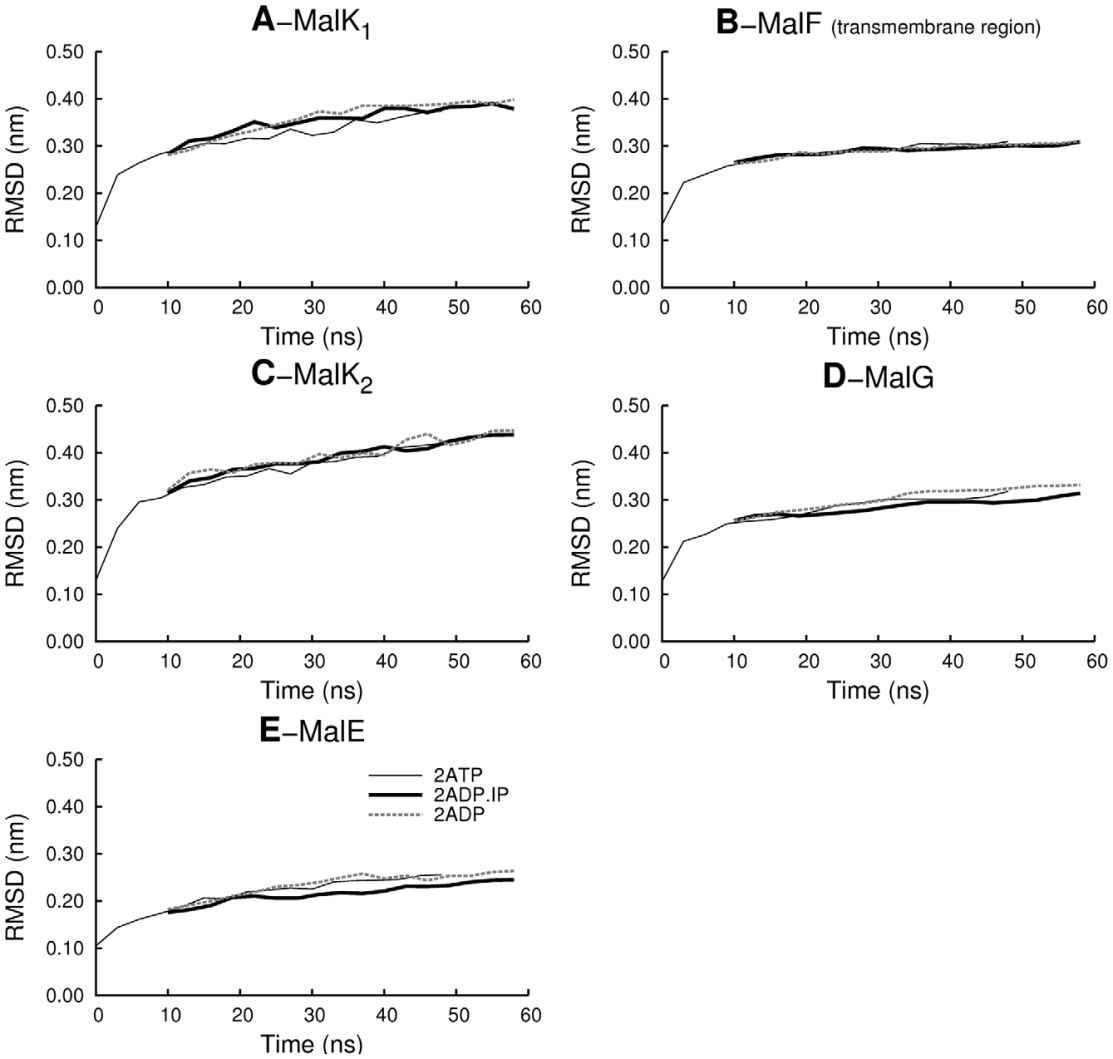
Conformational drift of the MalFGK_2_E complex relative to the X-ray structure. Cα RMSD evolution along the simulation time for (**A**) MalK_1_, (**B**) MalF transmembrane region. Due to a high amplitude rigid body rotation of the MalF periplasmic region (which will be debated in the following section), the RMSD was only determined for the MalF transmembrane region. (**C**) MalK_2_, (**D**) MalG and (**E**) MalE. The Cα RMSD was calculated relative to the X-ray structure and was determined after fitting each domain separately. Additionally, the RMSD values reported were averaged for all ten replicates for each state. Each point in the plot represents the average RMSD value for 100 ps.

The protein stability in our simulations was examined by visual inspection and by following the time evolution of several system properties, such as the root mean square deviation (RMSD) from the X-ray structure (see Figure 2) and the percentage of retained native secondary structure (SS) (Figure S4 in Text S1, section 4).

As can be seen in Figure 2, the RMSD profiles for all MalFGK_2_E components increased continuously in the **2ATP** state, reaching the global values of about 0.31 nm for the transmembrane domains (MalF and MalG) and 0.25 for MalE. The MalK domains exhibit more deviations from the X-ray structure as the Cα RMSD increases continually until it reaches values of 0.37 nm and 0.41 nm for MalK_1_ and MalK_2_, respectively. In general, the RMSD values reported, although large, are in the same order of magnitude as the ones reported in previous MD studies for other ABC transporters [49], [52], [58]. The evolution of the native SS content (see Figure S4 in Text S1, section 4) show that the overall secondary structure remains intact, with a secondary structure loss lower than 12% at the end the 50 ns simulation of the **2ATP** state. The RMSD values, together with the time evolution of native SS content, are indicative of structural stability for the simulated complexes.

The post-hydrolysis states simulated exhibit similar RMSD and native SS content evolution behavior to the one described for the **2ATP** state (note that in this figure all these states are compared with the X-ray structure, and not among themselves).

### Rigid body motion of the MalF periplasmic region

**Figure 3:**
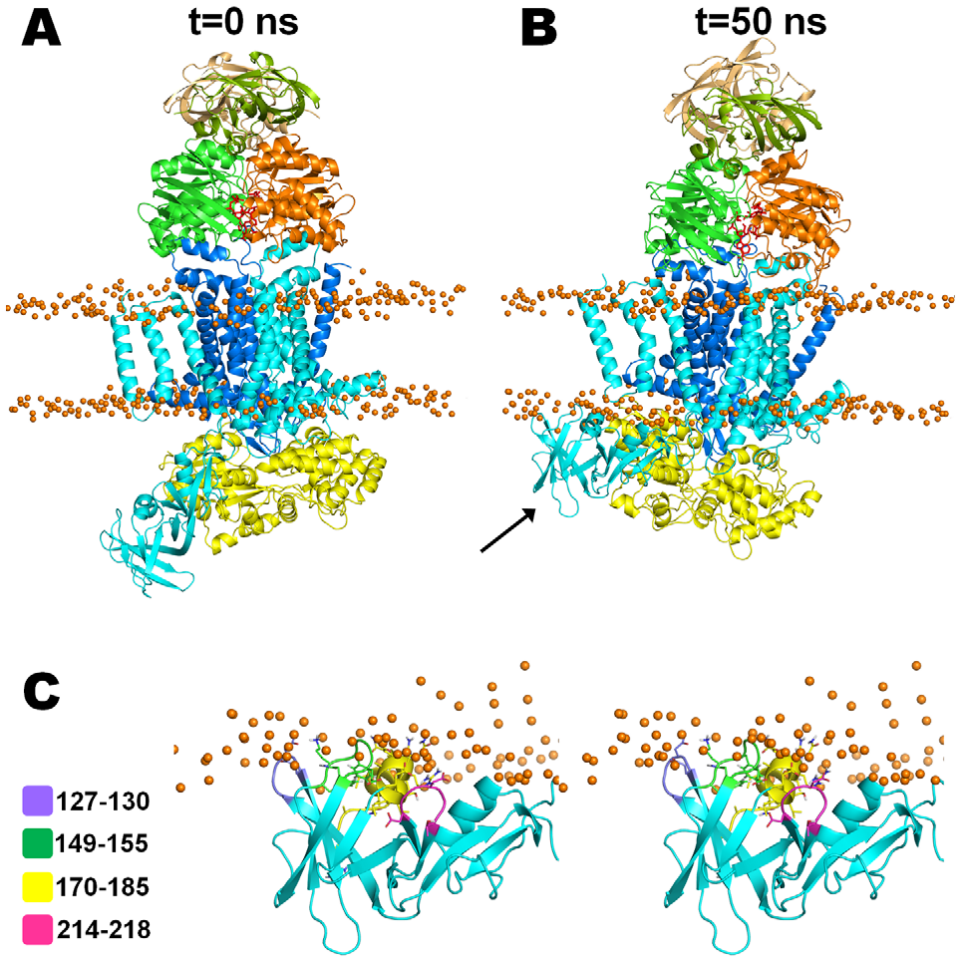
Detail view of the MalF-P_2_ region (residues 91–271) movement in the 2ATP replicate 3 simulation. MalK_1_, MalK_2_, MalF, MalG and MalE are colored is green, orange, cyan, blue and yellow, respectively. In these images, the regulatory domains are colored with lighter colors. ATP is shown as red sticks, whereas the phosphate atoms from the lipid head groups are represented as orange spheres. **A**- Snapshot at the beginning of the simulation. **B**- Structure obtained after 50 ns of simulation. The zone experiencing this rigid body movement is marked with a black arrow in both figures. **C**- Stereo detailed view of the MalF-P_2_ region and its new interactions with the lipid membrane. The residues that directly intereact with the membrane are colored in purple (res 127–130), green (res 149–155), yellow (res 170–185) and magenta (res 214–218), respectivelly.

In the first 20 ns of the **2ATP** simulations (in all 10 replicates) we observed a rigid body rotation of large amplitude towards the membrane, in the periplasmic region of MalF, namely in the MalF-P_2_ region (also designated by some authors as “MalF-P_2_ loop” [21]), which resulted in an high RMSD for this region (see Figure S5 in Text S1, section 5). However, the overall tri-dimensional fold of this transporter is maintained intact during the simulations (see Figure 3 for a representative example). After this large amplitude movement, the MalF-P_2_ region side chains establish new interactions with the membrane lipid head-groups. In the MalF domain (for all the replicates), the 127–130, 149–155, 170–185 and 214–218 segments are the regions that upon rotation, establish new interactions (such as H-bonds and van der Waals interactions) with the membrane (see Figure 3C for a detailed view). The 127–130, 149–155 and 214–218 segments are all situated in loop regions located between two anti-parallel β-sheets. The segment 170–185 in located in a short α-helix (helix49) that upon rotation becomes oriented roughly parallel to the membrane. Furthermore, together with the MalF-P_2_ region upward movement, the MalE N-terminal region is pulled toward MalG. The MalG-MalE approximation observed in our ATP simulations, is in agreement with experimental evidences reporting that, both MalF and MalG, are involved in MalE binding during the transport cycle [88], [89], a fact that was not observed in the crystallographic structure [21]. Biochemical and genetic studies suggested that the MalE N-terminal lobe interacts mainly with MalG, whereas the MalE C-terminal region is close to MalF during the transport cycle [88], [89]. So, in conclusion our simulations seem to capture the functional details of this ABC transporter.

At this stage, we are inclined to believe that the MalF-P_2_ region conformational change is a natural adaptation of this region to the membrane, which may be different from the crystallographic environment. If we analyze the crystallographic contacts between the MalF-P_2_ region and the other molecules in the crystal, we see that it interacts strongly with two symmetry mates. Therefore, its exact conformation in the crystal may be strongly determined by these contacts, and be different from the structure of the transporter within a membrane.

### MalK dimer interface

**Figure 4:**
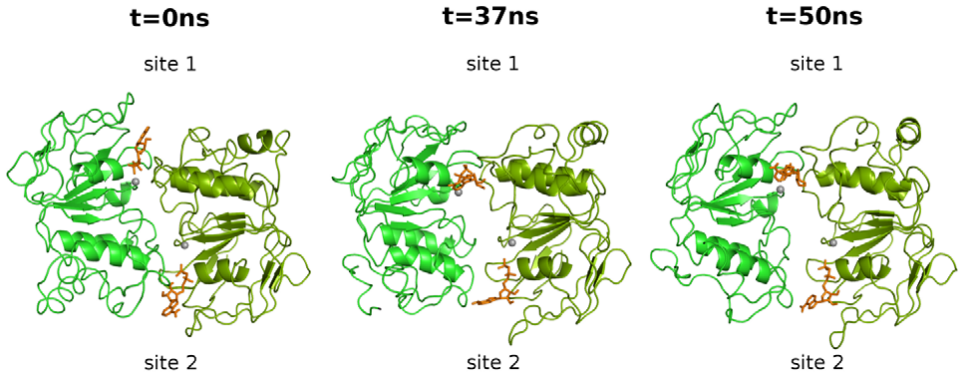
Snapshots of the MalK dimer opening in the 2ADP state for replicate 8. The left side image was obtained at 0 ns, whereas the middle represents the beginning of the dimer dissociation (after 37 ns). The right side image corresponds to the final structure (after 50 ns). The MalK monomers are colored in green, ADP is represented with orange stick and Mg with gray spheres. For clarity purposes, the regulatory domains are not represented in these three images. For a clearer visualization of the dimer interface opening, we suggest the visualization of the animation (Video S1) included in Text S1, section 9.

In a previous MD simulation work, Wen and Tajkhorshid [45], using the isolated NBDs subunits of the maltose transporter (MalK) in solution, were able to observe the atomic details of the MalK interface opening upon ATP hydrolysis, in a timescale ranging from 30 to 50 ns. In order to see if the MalK dimer dissociation was observable in our complete transporter simulations, the distance between the P-loop of one MalK monomer and the ABC signature motif of the other MalK monomer was determined for all states (see Figure S6 in Text S1, section 6). From this measurement, it is evident that in the **2ATP** and in the **2ADP.IP** states, no MalK dimer interface separation was detected in all ten replicates, within the 50 ns simulated, evidencing that there was no dissociation of the MalK dimer. However, in the **2ADP** state, in one of the ten replicates (replicate 8), the dissociation of the MalK interface, in binding site 2, was observed after 37 ns of MD simulation (see Figure 4 and Figure S6 in Text S1, section 6). In this replicate, at the beginning of the simulation, both binding sites are closed and the two ADP molecules are bound at the interface between monomers. At the end of the simulation, binding site 2 is completely separated and the nucleotide is only bound to the P-loop residues. This observation seem to agree with the experimental evidences showing that, in the MalK dimer, ADP, unlike ATP, cannot promote ATPase dimerization nor the stabilization of the closed dimeric form [37].

**Figure 5:**
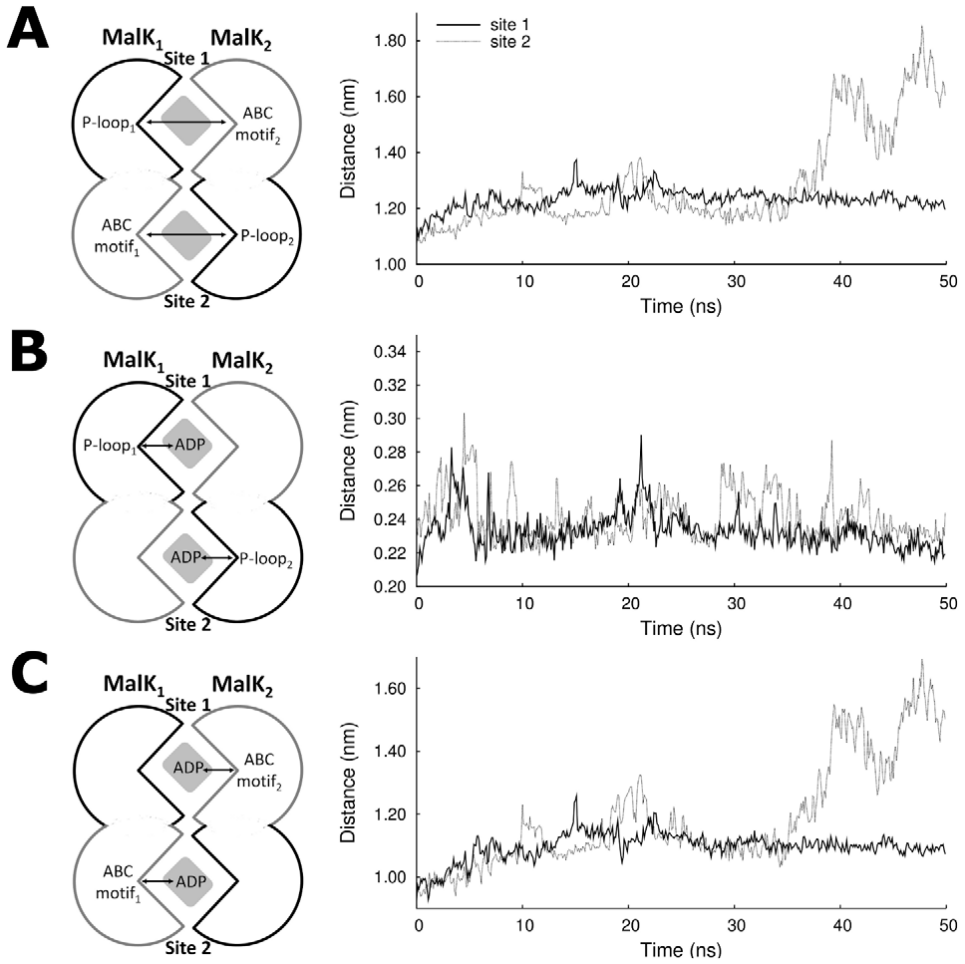
Opening of the MalK interface in the 2ADP state (replicate 8). The black and gray lines correspond to distances measured in binding site 1 (formed by the MalK_1_ P-loop and the MalK_2_ ABC motif) and 2 (formed by the MalK_2_ P-loop and the MalK_1_ ABC motif), respectively. Each point represents the average value over 100 ps. **A**) Distance between the P-loop motif of one MalK monomer and the ABC signature motif of the other. **B**) Distance between the ADP α,β-phosphate and the P-loop residues. **C**) Distance between the ADP α,β-phosphate and the ABC signature motif residues.

In the ADP-bound state, with the objective of analyzing with more detail the MalK interface dissociation and the rearrangements involved in this process, several distances were measured for replicate 8. To quantify dimer separation, we determined the distance between the P-loop and the ABC signature motif forming the binding sites (Figure 5A). Additionally, the distances between the ADP nucleotide and the P-loop (Figure 5B), and the ABC signature motif (Figure 5C), were also determined.

As can be seen in Figure 5A, during the MalK interface dissociation, the distance between the binding site residues increases ∼0.44 nm in binding site 2, whereas in binding site 1 this distance does not change considerably. Moreover, the interface opening is accompanied by the dissociation of the ADP phosphate atoms from the ABC signature motif (Figure 5C), although they did not dissociate from their corresponding P-loop residues (Figure 5B), preventing the nucleotide diffusing away from the binding site. Similar observations for NBD dimer dissociation were reported for the simulations of isolated MalK dimer [45] and for the ADP.IP-bound Sav1866 exporter [39]. Surprisingly, and although ADP is present in the two binding sites, only site 2 presents a clear opening of the MalK interface. This asymmetric movement, observed in previous theoretical works (not only in the MalK dimer [45], but also for several other ABC members [39], [46], [47]), is frequently attributed to the stochastic nature of the opening process. This cannot be clarified here, since we have only one event in the ten replicates simulated.

Despite this considerable motion in the NBDs, the effect on the transmembrane domains is very limited, and no major conformational changes were observed in MalF and MalG, within the simulated timescale (see Figure S7 in Text S1, section 6).

### Structural differences during the ATP-cycle in the MalFGK_2_E complex

**Figure 6:**
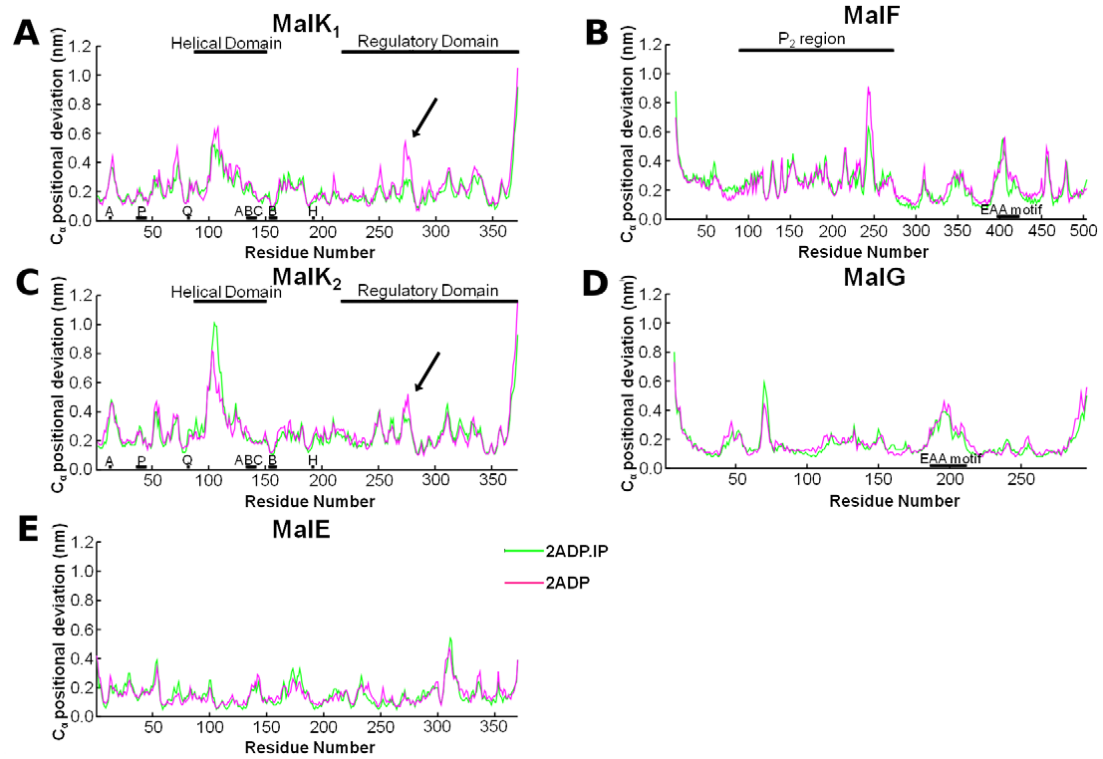
Average Cα-Positional Deviation (over the last 10 ns) with respect to the average 2ATP conformation. The deviation values displayed were obtained by comparing the average structure of each replicate with the average **2ATP** structure for the same replicate, in order to eliminate the structural differences arising from the natural variation between replicates (for more details about this methodology see [38]). The individual differences were averaged afterwards over all ten replicates. The relevant structural family motifs are marked in the plots: P-loop (P), Q-loop (Q), ABC signature motif (ABC), Walker-B motif (B), the H-motif (H) and the “EAA” motif (where the Coupling Helix is located [20]). In figure **A** and **C**, the black arrows identify the segment ranging from residue 271 to 276, which undergoes a significant rearrangement after IP exit. Average C_α_ Positional Deviation for **A**-MalK_1_, **B**- MalF, **C**- MalK_2_, **D**-MalG and **E**-MalE.

It is nowadays accepted that the ATP-cycle (nucleotide binding/hydrolysis and release of its products) induces conformational changes in the ATPase domains, which are then transmitted to the transmembrane region [26], [29]. In order to identify the hydrolysis-dependent conformational changes and the residues involved in inter-domain communication, the Cα positional deviation (relative to the **2ATP** state) was calculated for both post-hydrolysis states (Figure 6). These deviations were determined as a function of the residue number, for the last 10 ns of simulation, using a methodology previously described for the identification of the hydrolysis-dependent conformational rearrangements in the isolated MJ0796 NBD dimer [38].

Upon analysis of Figures 6A and 6C, we can observe that, although the regions showing the largest conformational changes are generally the same in the two MalK monomers, the amplitude of these changes is significantly different between monomers. The clearest example of this MalK asymmetric behavior is the HD region, where the conformational changes are more pronounced in the MalK_2_ monomer. This uneven behavior has already been reported in previous MD works, not only in the MalK dimer [43], [45], but also in other ABC members [38], [39], [44], [48], [50], [53], [56], [57]. At this stage, we are still unable to conclude whether this asymmetry arises from the X-ray structure itself (due, for example, to different crystallographic contacts) or if it is an intrinsic characteristic necessary for the functioning of this protein. In the present case, the MalK asymmetry can also arise from the effect exerted by the TMDs in the ATPase domains, which, contrary to the NBDs, are two different membrane proteins. For MalK_1_ and MalK_2_, the segments 14–15, 99–112 and the C-terminal (residues 366–371) can be identified as the regions presenting relevant differences between the two post-hydrolysis and the pre-hydrolysis state. In general, the regions presenting higher Cα positional deviation values are the same for **2ADP.IP** and **2ADP**. However, one exception was detected in the segment 271–276 (marked by a black arrow in Figures 6A and 6C), which undergoes significant rearrangement only after IP exit. For the transmembrane domains, MalF and MalG (Figures 6B and 6D), the major conformational deviations, relative to the **2ATP** state, are concentrated in very narrow and specific regions. In MalF, the zones presenting relevant rearrangements during the ATP-cycle are the segments 240–249 and 402–407, and the N-terminal. In MalG, the residues with higher positional deviations are the 70–72 and 190–197 segments, the N-terminal and the C-terminal regions. Finally, and based on the low average positional deviation values observed in Figure 6E, MalE seems to be very little affected by nucleotide hydrolysis and IP exit (with the exception of the residues ranging from 310–311), at least in the 50 ns timescale.

**Figure 7:**
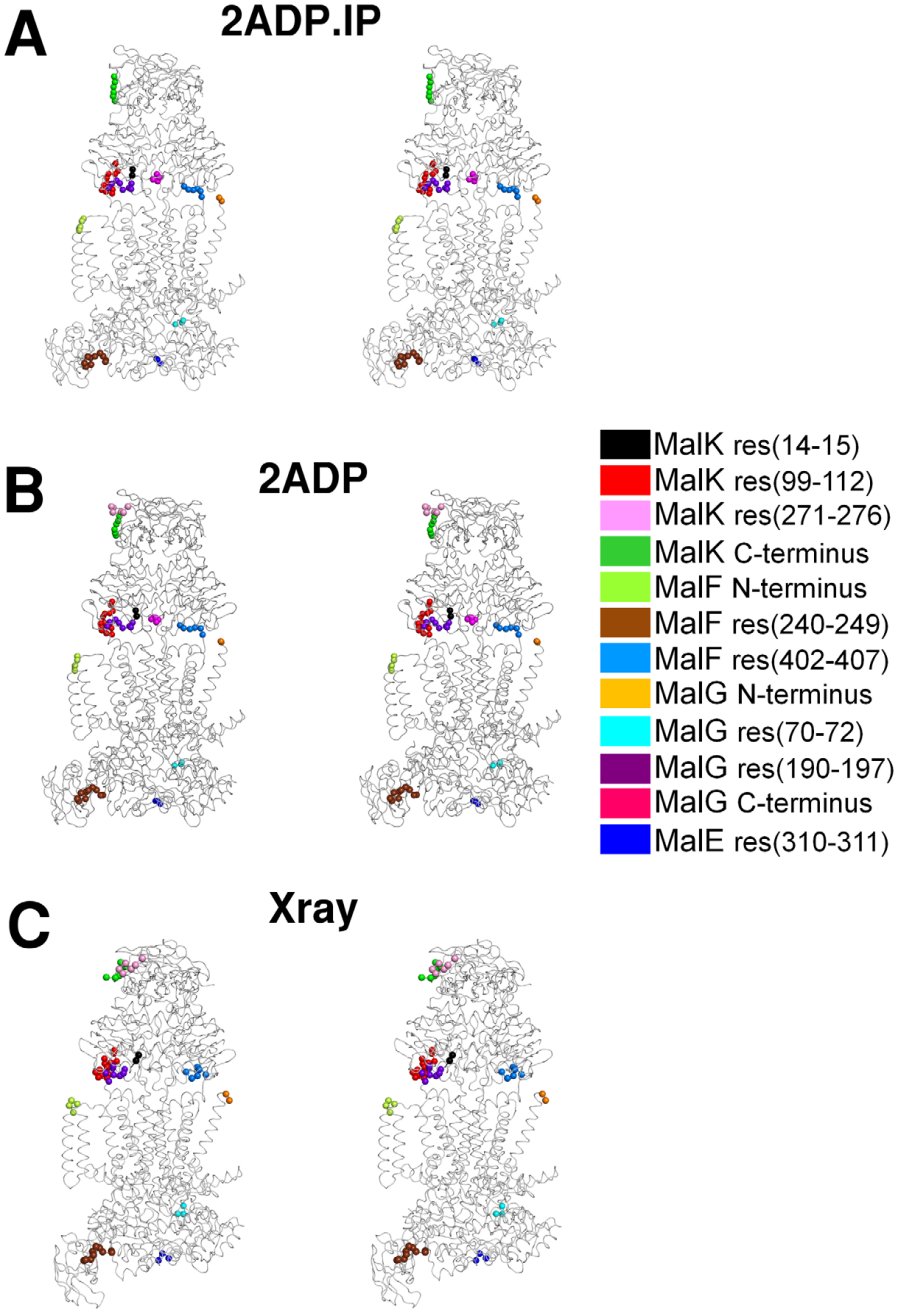
Stereo image of the A) global average structure for the 2ADP.IP state , B) global average structure for the 2ADP state. C) X-ray structure. The global average structures were determined from the last 10 ns of simulation, over all ten replicates, after fitting to the X-ray structure. The regions presenting the most relevant positional deviations, relative to the average **2ATP** state, are highlighted in different colors (see the legend). For clarity, only the MalK_1_ segments are colored, but similar positional deviations are also observed in the same MalK_2_ segments.

In order to identify the spatial organization of the residues undergoing the most relevant displacements during the ATP-cycle, the position of these residues were mapped in the average structure, both for the **2ADP.IP** and the **2ADP** states (Figure 7).

From Figures 6A, 6C and 7, one of the major conformational changes observed in the NBD region is located in the 14–15 segment (also named A-loop). The A-loop is located in the external region of the MalK monomer, connecting two β-sheets (Figures S1 and S2 in Text S1). This region was named A-loop due to presence of an essential conserved aromatic residue, which is known to interact with the nucleotide adenine ring during the ATP cycle [90]. Our previous work [38], performed in the isolated ATPase dimer from *Methanococcus jannaschii*, has also identified the A-loop segment as one of the regions showing major conformational changes during the ATP-cycle [38]. The other MalK region evidencing high Cα deviation is the HD region (residues 99–112). This region is located in an external and flexible region of the MalK monomer, and is in direct contact with the transmembrane domains (MalF and MalG), more specifically with the coupling helices. This is the region that was identified by ourselves [38], [48] and others [41], [44], [47] in previous studies in isolated NBD dimers to be particularly sensitive to changes in the nucleotide bound state. Recently, using the complete ABC exporter from *Staphylococcus aureaus*, we also identify this region as the one displaying more pronounced rearrangements upon ATP hydrolysis [39]. These modeling works come to confirm, from a structural perspective, previous experimental data [16], [91]–[93] which have shown the HD as one of the regions more affected by the ATP-hydrolytic cycle and probably responsible for inter-domain communication, providing the link for energy transmission from the NBDs to the TMDs [94]. In our simulations, we could determine a small amplitude rotation of the HD upon hydrolysis (see Figure S8 in Text S1, section 7), although the observed rotation is significantly smaller than the ones previously described in simulation works performed on the isolated NBD dimers [38], [44], [47] or inferred from X-ray analysis [16], [36], [37], [91]–[93]. This difference may be related with the timescale of our simulations and/or to the effect exerted by the TMDs (especially the “EAA motif”) in the HD region. It is also possible that the absence of the TMDs in previous simulation work allowed the observation of non-physiological, higher amplitude, movements in the HD.

In MalF (see Figures 6B and 7), two segments are mostly affected by nucleotide hydrolysis and IP exit. The first segment is formed by residues 240–249 and it is located in the MalF-P_2_ region, directly contacting MalE. The second segment comprises residues 402–407 and is located in the “EAA” motif, more specifically in the coupling helix region. The “EAA” motif (EAA-X(3)-G) [95], [96] is the direct contact point between MalK and MalF/G, and it is formed by two short cytoplasmic helices oriented roughly parallel to the membrane plane [21]. One of these helices, named Coupling Helix [20], docks directly into a cleft in the helical sub-domain region of MalK [21], providing the bulk of the interdomain contacts. Despite the limited sequence similarities between the TMDs region in the ABC family [97], other ABC members (such as the Sav1866 exporter [25] and the HI1470/71 importer [19]) also present coupling helices located in similar regions, and directly interacting with the ATPase domains. Moreover, upon comparison of the results obtained for the complete MalFGK_2_E importer and for the complete Sav1866 exporter [39], we were able to see that, for both transporters, the coupling helices are one of the regions presenting major conformational changes during the ATP-cycle, despite their high sequence variability. It now becoming clear that these helices have an essential role in the mechanism of conformational changes transmission between domains, as suggested by Locher and Dawson [25].

In MalG (Figures 6D and 7), the regions showing major conformational changes during the ATP-cycle are the residues 70–72, 190–192 and the C-terminus. The first set of residues is located in the periplasmic side of the membrane, in direct contact with MalE, while the second set is located in the “EAA” motif, in the coupling helix region (similarly to MalF). During the ATP-cycle, the two coupling helices (one in MalF and the other in MalG), although different in sequence, are both similarly affected by the corresponding MalK HD rearrangements. Lastly, the MalG C-terminal region is also highly influenced by the ATP-cycle, mostly due to its location, since it is inserted into the MalK dimer interface, close to the Q-loop motifs (close enough to form some hydrogen bonds with the Q-loop residues) [21]. It was also suggested that, although the MalG tail is not essential for the NBDs dimer formation [36], the interactions formed between these two domains contribute for the Q-loop ordering, and may be import for the intermediate states of the transport cycle [21]. The Q-loop motif, and especially, the conserved glutamine residue located in this region, has long been suggested to play a key role in hydrolysis, by coordinating the magnesium ion located in the binding site, and by orienting the nucleophilic water needed for ATP hydrolysis [16].

### Maltose position during ATP hydrolysis

In the X-ray structure, the maltose binding site is located at the base of the transmembrane cavity, approximately halfway across the membrane and it is formed exclusively by MalF residues [21]. Maltose is bound by hydrogen bonds and by ring stacking interactions to several MalF aromatic residues [21]. During our simulations, the maltose position and the allocrite binding site rearrangements were monitored in order to understand if hydrolysis and the presence of IP directly influences the binding site conformation, and consequently the allocrite affinity for this site. This was done for all replicates of the **2ATP**, **2ADP.IP** and the **2ADP** states. For the majority of the replicates, maltose does not move away from the binding site (see Figure S9 in Text S1, section 8), but there are two exceptions, one in the **2ATP** (replicate 2) and another in the **2ADP.IP** (replicate 1) simulations. In these two cases, the interactions with the MalF binding site residues (see Figure S10 in Text S1, section 8), mainly the hydrogen bonds and the π-π interactions with the aromatic residues, are severely reduced (data not shown), which enables maltose to move laterally and exit from the binding site, interacting with some MalG residues (as for example, glutamine 129 and glutamate 229). Nevertheless, in all replicates, no significant change was observed during the 50 ns of simulation in the overall shape of the transmembrane cavity and in the maltose position between the three distinct states (see Figure S11 in Text S1, section 8).

### Concluding remarks

Although ABC transporters have been widely studied during the last thirty years, the understanding of this family and its transport mechanism is still very incomplete. In particular, and although it is clear that the energy arising from ATP hydrolysis is propagated from the NBDs (namely from the nucleotide binding pockets) to the TMDs, the details of this communication mechanism are still not fully understood. In this study, we have performed extensive MD simulations of a the model import system (MalFGK_2_E from E.*coli*) in three distinct states of the ATP-cycle in order to provide new relevant insights into the inter-domain communication mechanism and into the conformational rearrangements induced by the ATP-hydrolytic cycle. Nucleotide hydrolysis and IP exit induces major conformational rearrangements in specific residues or segments, both in the ATPase domains (MalK) and in the transmembrane regions (MalF and MalG). The segments presenting major rearrangements are the A-loop and the HD region, for MalK, and the “EAA motif” region and the coupling helix, for both MalF and MalG. Moreover, from the MD simulations performed here for the MalFGK_2_E importer together with the MD simulations reported previously for the Sav1866 exporter [39], we are now able to identify the HD region and the “coupling helices” as the “key players” of the inter-domain communication in the ABC family. Given their importance in the simulations, we think that the residues forming the coupling helices, the HD and the A-loop are excellent candidates for mutation experiments in order to verify their importance. Moreover, in order to clarify the physical reality behind the observed rigid body rotation of the MalF-P_2_ region, it could be interesting to determine the distance (for example, by site-specific chemical cross-linking experiments like [98]) between possible pairs of residues located in MalG (for example, in α-helice 70) and in the Mal-P_2_ region (for example, in α-helice 49). Given that the A-loop is also a zone that suffers substantial conformational changes in the ATP cycle, residues in this zone would also be good candidates for mutation, specially W13 of MalK, which is a conserved residue in ABC transporters, and one that has already been mutated in other members of this family, impairing or reducing transporter function [90].

Additionally, in the post-IP exit state studied, we were also able to observe (in one replicate) the MalK dimer interface dissociation in one of the two binding sites. In this replicate, and similarly to what was described in other simulation works [39], [45], [47], the interface opening was accompanied by the dissociation of ADP from the ABC signature motif, but not from the P-loop motif. However and surprisingly, the opening of one of the nucleotide binding sites did not significantly alter the MalF and MalG (the TM domains) conformations or the allocrite binding site, at least in our simulated timescale.

Although it is clear that many significant questions remain with respect to the ABC transporter family, over the last years, molecular dynamics simulations (performed both in complete importers [50], [51], [53], [54], [57], [59], and exporters [39], [49], [52], [56], [58], [60] as well as in the isolated NBD dimers [38], [42], [44], [45], [47]) have proven to be a powerful tool for studying ABC transporters and have significantly contributed with new insights into the understanding of their mechanisms. A general mechanism for coupling hydrolysis and energy transduction to allocrite translocation (independently of the transporter directionality) in ABC transporters is now starting to be unraveled and its “key players” are the HD region and the TMD coupling helices.

## Supporting Information

Text S1 Supporting Information Text S1. This file includes 9 distinct sections: 1- Protonation state of protonable residues. 2- DMPC bilayer construction. 3- Outline of the MD simulations performed. 4- MalFGK_2_E structural stability. 5- Rigid body motion of the MalF Periplasmic region. 6- MalK dimer interface. 7- Helical sub-domain rotation. 8- The position of maltose during ATP hydrolysis. 9- Movie of the MalK dimer interface opening in the **2ADP** state.
(DOC)

Video S1 MalK dimer interface opening for replicate 8 of the **2ADP** state.
(AVI)
