# Supplementary material for: Inter-domain Communication Mechanisms in an ABC Importer: A Molecular Dynamics Study of the MalFGK2E Complex
Source: PLoS Comput Biol. 2011 Aug 4;7(8):e1002128. doi: 10.1371/journal.pcbi.1002128 (PMC3150292; doi:10.1371/journal.pcbi.1002128)
Supplement: Text S1 — Supporting Information Text S1. This file includes 9 distinct sections: 1- Protonation state of protonable residues. 2- DMPC bilayer construction. 3- Outline of the MD simulations performed. 4- MalFGK2E structural stability. 5- Rigid body motion of the MalF Periplasmic region. 6- MalK dimer interface. 7- Helical sub-domain rotation. 8- The position of maltose during ATP hydrolysis. 9- Movie of the MalK dimer interface opening in the 2ADP state. (DOC) [file pcbi.1002128.s001.doc]

**SUPPLEMENTARY MATERIAL**

**1. Protonation state of protonable residues**

The protonation state of each individual protonatable group (residues and nucleotide species) at the pH of interest (pH=7.0), has to be specified prior to the MD simulations. This was determined using methodologies for studying the thermodynamics of proton binding, which are described elsewhere . These methodologies use a combination of Poisson-Boltzmann calculations, performed with the package MEAD (version 2.2.5) , and Metropolis Monte Carlo simulations, using the program PETIT (version 1.3) . Since the maltose transporter is a membrane protein with an asymmetrical transmembrane pore, a custom made model was build for the calculations with this system (see figure S1). In this model, the membrane was introduced in the x-y plane as a low dielectric media formed by tree different regions. The first and third regions represent the hydrophilic lipid head groups, and are 7.5 Å wide each. The second region, with a thickness of 26 Å, characterizes the middle of the bilayer and models the hydrophobic tails of the lipids. Different values of dielectric constant were assigned, based in the atomic z coordinates: for the first and third slabs of the bilayer the dielectric constant used was 20, while for the middle of the bilayer the dielectric constant used was 4. All points located inside the transmembrane pathway were treated with the dielectric constant of the solvent (80).


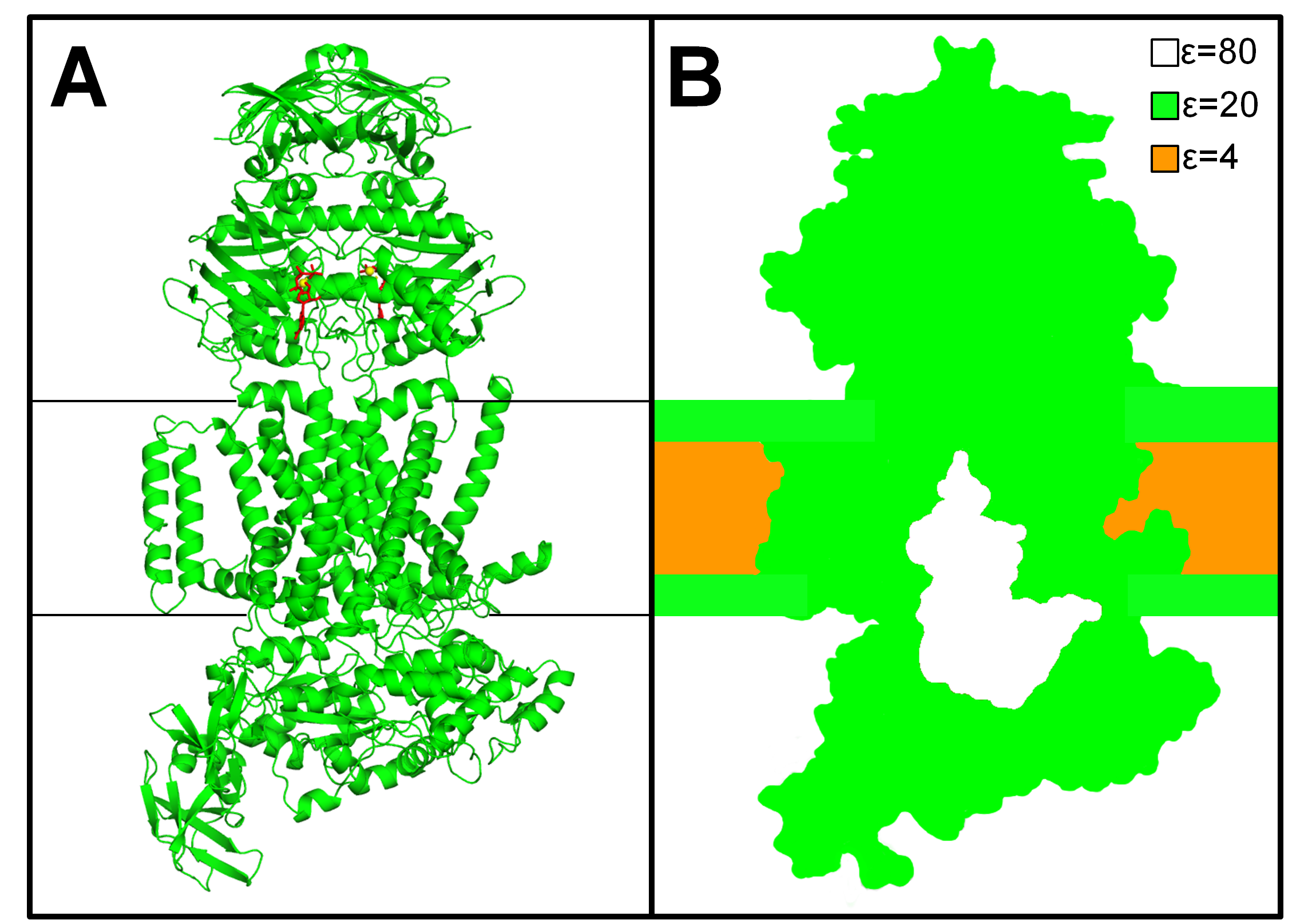


**Figure S1**- A schematic view of the model used to calculate the protonation states with the program MEAD (version 2.2.5) and PETIT (version 1.3) for the MalFGK_2_E complex inserted in a lipid bilayer. **A**- Overview of the crystal structure of the complex formed by the ABC importer MalFGK_2_ and the maltose binding protein (MalE). The protein is rendered as a cartoon, the ATP molecules are represented by red sticks and the magnesium ions as yellow spheres. **B**- Overview of the complete model for the MalFGK_2_E complex built by the package MEAD. The membrane is represented by three slabs of low dielectric media. The first and third slabs models the hydrophilic lipid head groups of each leaflet of the bilayer (ε=20), and the middle slab (coloured in orange) represents the lipid hydrophobic tails (ε=4). In this model, the slabs representing the hydrophilic parts of the lipids are 7.5 Å wide, whereas the slab representing the lipid tails is 26 Å wide.

According to our calculations, all glutamic and aspartic acids are deprotonated with the exception of MalG Glu149. Furthermore, all lysines and arginines are protonated with the exception of MalF Lys16, Lys61 and Lys314 and MalG Lys90, Lys118 and Lys123. All free cysteines are protonated and the protonation state of all histidines is described in table S1:

**Table S1-** Protonation state for all the histidine residues of the MalFGK_2_E complex. HisA refers to the histidine residue with a zero total charge and protonated at the Nδ position, HisB corresponds also to a neutral residue but in this case protonated at Nε. The HisH represents the protonated histidine at both Nδ and Nε with the overall charge of +1.

| **MalG** | |  | **MalK** | |
| --- | --- | --- | --- | --- |
| **Residue Number** | **Protonation state** |  | **Residue Number** | **Protonation state** |
| 17 | HisA |  | 27 | HisH |
| 58 | HisH |  | 89 | HisB |
| 159 | HisB |  | 125 | HisB |
| 173 | HisB |  | 180 | HisH |
|  |  |  | 192 | HisB |
| **MalE** | |  | 223 | HisB |
| **Residue Number** | **Protonation state** |  | 289 | HisB |
| 39 | HisH |  | 317 | HisB |
| 64 | HisB |  | 353 | HisA |
| 203 | HisH |  | 366 | HisH |

For MalK, MalF and MalG, the N- and C-terminals were considered neutral (NH_2_), since the crystal structure lacked some of the first and last residues. For MalE, according to our calculations, the N-terminal was positively charged (NH_3_^+^), whereas the C-terminal was negatively charged (COO^-^).

**2. DMPC bilayer construction**

**128 DMPC bilayer**

An initial model for a hydrated 128 DMPC bilayer (64 lipids in each leaflet and 4214 water molecules), obtained from the website of Peter Tieleman’s group at http://moose.bio.ucalgary.ca, was used as a starting point for the construction of our DMPC membrane. The water/lipid content was about 33 water molecules per lipid.

Since the initial membrane model had been equilibrated under different simulation conditions, we submitted the 128 DMPC molecules for 30 ns of MD simulation. The simulation was performed using the GROMACS 3.3.1 package . The lipid parameters used were based on the 53A6 GROMOS96 force field , except for the atomic partial charges, which had been previously derived by Chiu and coworkers . The membrane equilibration was performed at constant temperature conditions (310K). This temperature is above the phase transition temperature reported in literature for DMPC (Tm=296-297K) in order to ensure that the bilayer is in the liquid crystalline state . The setup for the molecular dynamics simulations is similar to the one reported in the main article in the Materials and Methods section.

The evolution for some lipid properties (area and volume per lipid) along the simulation time can be followed in figure S2.


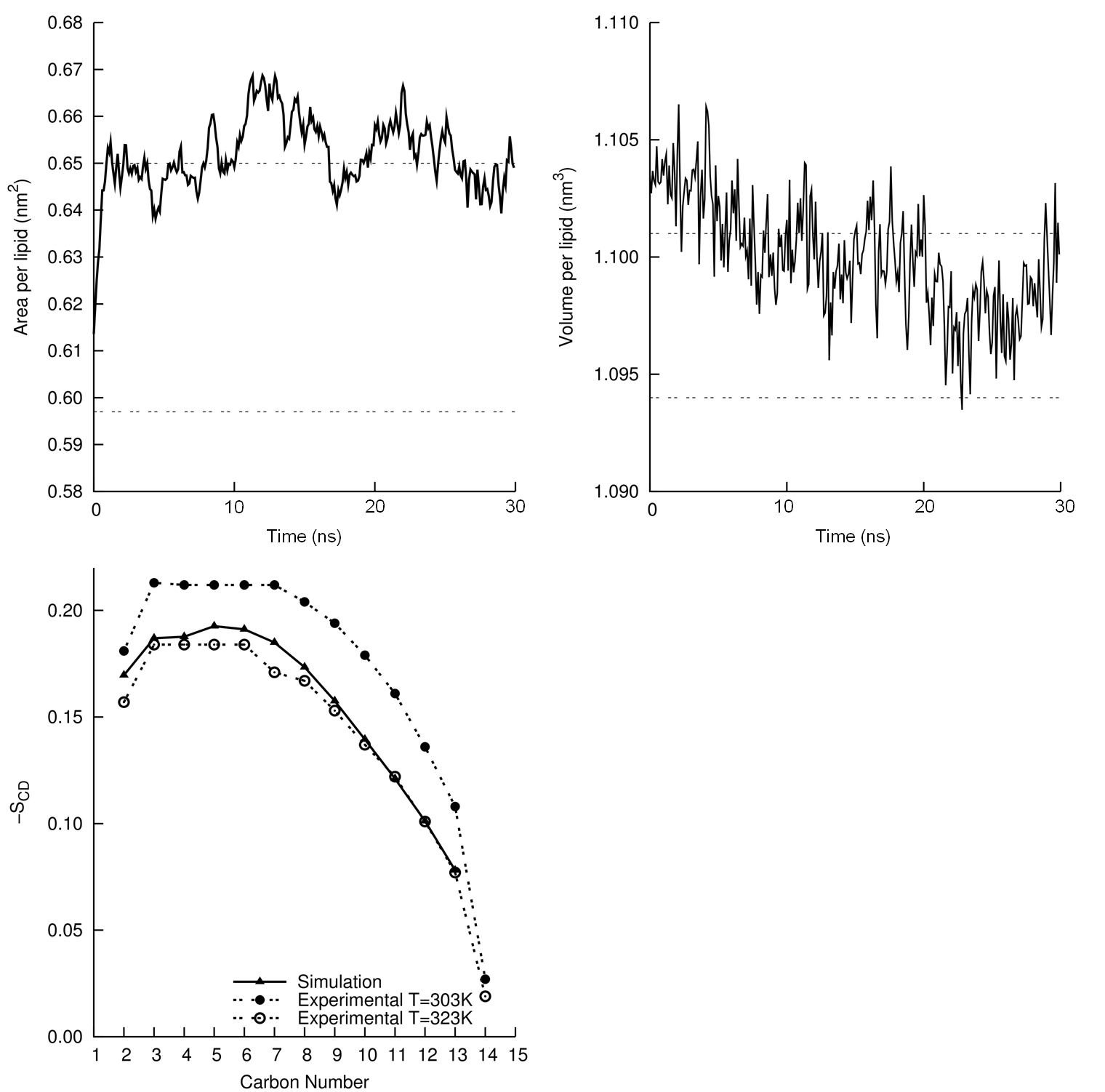


**Figure S2- A-** Area per lipid for the 128 DMPC as a function of the simulation time (solid black line). The horizontal dashed lines represent the upper and lower limit ranges for the experimental values . **B**- Time evolution of the volume per lipid (solid black line). Again, the horizontal dashed lines represent the upper and lower limit ranges for the experimental values . In both plots, each point represents the average value over 100 ps. **C**- Deuterium order parameters. Black and white circles are the experimental results averaged over both sn-1 and sn-2 chains for two different temperatures (T=303K and T=323K), while the black triangles represent the data obtained from the present simulation.

As can be seen, in the end of the 30 ns of simulation, the area (figure S2A) and volume per lipid (figure S2B), despite showing some fluctuations, remain more or less within the experimental range. Based on previous works with DMPC lipidic bilayers , we considered the first 25 ns as an equilibration period, and this data was discarded. The average values for the area and volume per lipid over the last 5 ns of simulation are 0.648 nm^2^ and 1.098 nm^3^, respectively. Additionally, the organization of the hydrocarbon tails can be calculated from the simulation and compared to experimental results obtained using NMR techniques (figure S2C). As can be seen in figure S2C, the values for the deuterium order parameters from the simulations are in good agreement with the values obtained experimentally .

**512 DMPC bilayer**

The 512DMPC bilayer was generated by the replication of the already described pre-equilibrated 128 DMPC bilayer, in both the x and y directions. The resulting system was then energy minimized and re-equilibrated again for over 20 ns. The system was subjected to 5000 steps of steepest descent energy minimization in order to remove excessive strain, and then submitted to 100 ps of restrained MD simulation: the first 20 ps with restraints in all heavy atoms, including water (force constant 1000 kJ mol^-1^ nm^-2^), followed by 30 ps with restraints in all lipid heavy atoms (force constant 1000 kJ mol^-1^ nm^-2^) and the last 50 ps with restraints in all the lipid heavy atoms, but in this case with a lower value for the force restraints (5 kJ mol^-1^ nm^-2^). The unrestrained MD simulations started from this point forward and lasted 20 ns. All the simulation details were exactly like the ones described in the main article in the Materials and Methods section. In the end of the re-equilibration period, similarly to the 128 DMPC bilayer, the 512DMPC bilayer presents area and volume per lipid values within the experimental range. The average value for the area and volume per lipid over the last 5 ns of simulation is 0.658 nm^2^ and 1.099 nm^3^, respectively.

**3. Outline of the MD simulations performed.**

**
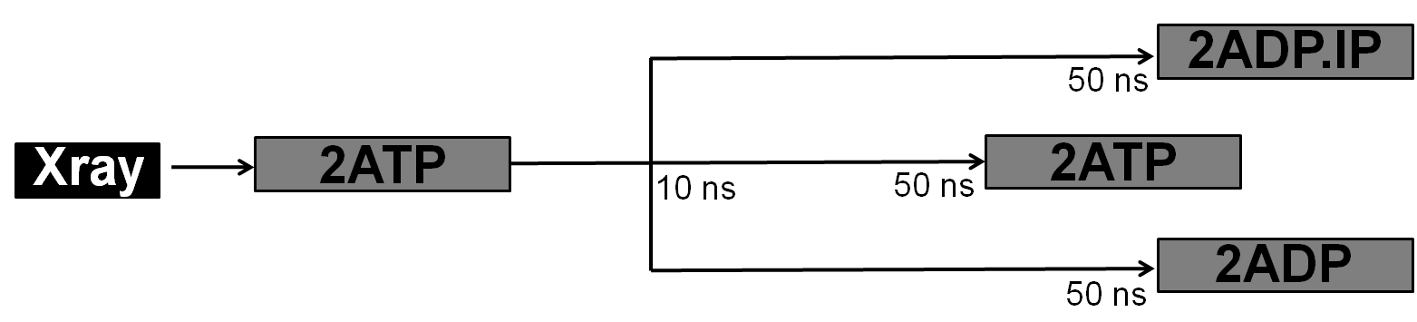
**

**Figure S3- Outline of the MD simulations performed.** Starting from the X-ray structure , the MalFGK_2_E complex with MgATP bound in the binding sites was reconstructed and the E159Q mutation reverted in order to obtain the wild type complex. Then, the ATP-bound state (**2ATP**) was simulated for a period of 50 ns. After 10 ns of simulation of the **2ATP** state, two new states were prepared by replacing the ATP nucleotides by ADP (**2ADP**) or ADP and IP (**2ADP.IP**). Ten replicates, 50 ns each, were calculated for all the three states reported. This figure represents the procedure used for only one replicate.

**4. MalFGK_2_E structural stability**

For all states (**2ATP,** **2ADP.IP** and **2ADP**), the protein behaviour during the 50 ns of simulation was carefully examined for all ten replicates, by the determination of the root mean square deviation from the X-ray structure (see figure 2 of the article) and the secondary structure content (figure S4). In general, the RMSD values reported for our three states, although large, are in the same order of magnitude as the ones reported in previous MD studies for other ABC transporters . Additionally, the percentage of secondary structure content (averaged over the ten replicates) was determined over the simulation time in order to see if abnormal structure loss was observable (figure S4).


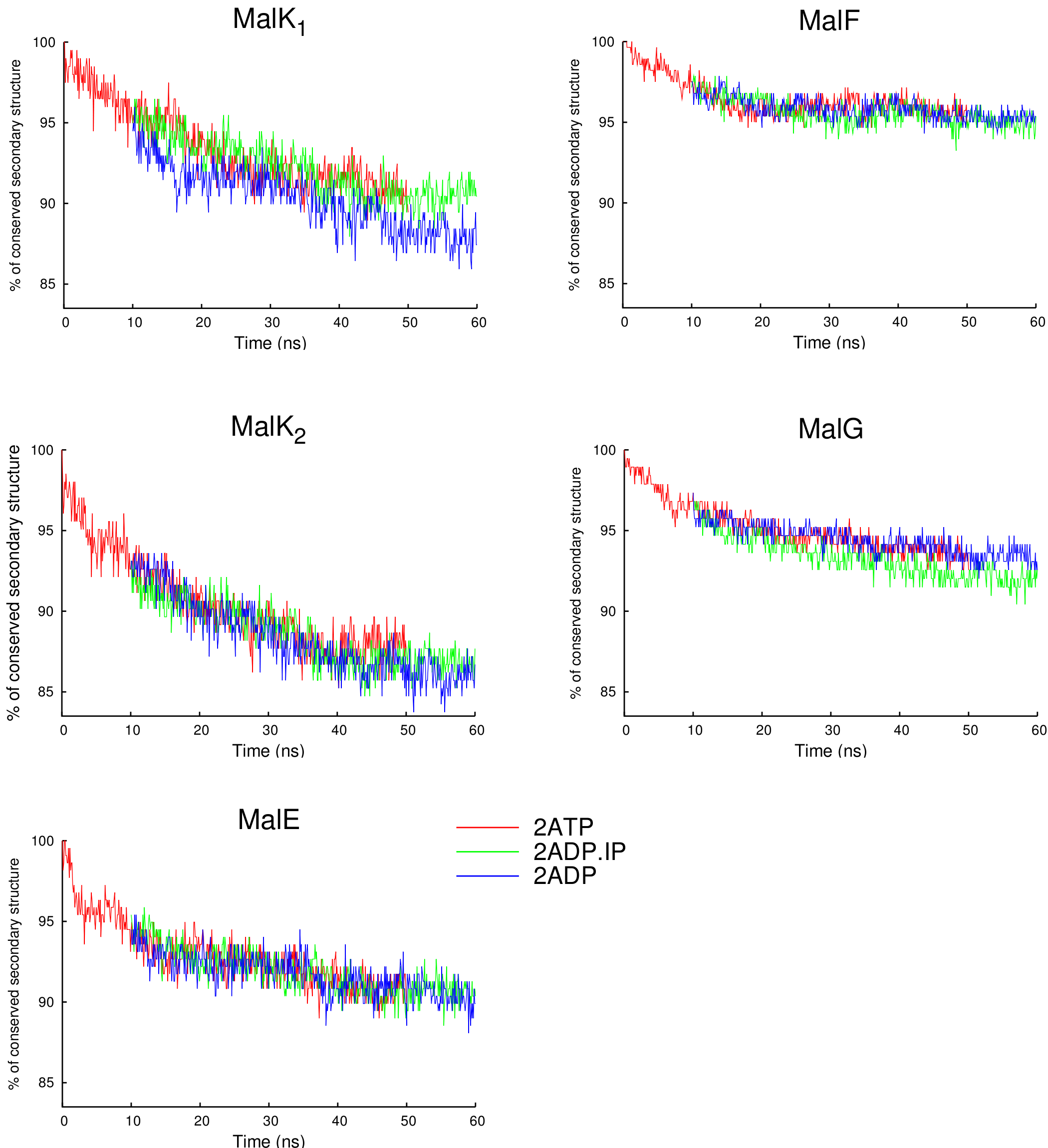


**Figure S4**- Percentage of residues within the same secondary structure class as in the X-ray structure (calculated using DSSP ) for MalK_1_, MalK_2_, MalF, MalG and MalE for the **2ATP** (red line), **2ADP.IP** (green line) and the **2ADP** (blue line) states. The averages were obtained over all 10 replicates for each state. The regular secondary structures considered for this measure were the α-helix, the β-sheet, the 3_10_ helix and the β-bridge (DSSP classification ).

As can be seen from figure S4, the overall secondary structure pattern is maintained, for both the pre- and post- hydrolysis simulated states, and only a small (<12%) secondary structure loss was measured. The RMSD values, together with the time evolution of native secondary structure content, are indicative of structural stability for the MalFGK_2_E complex.

**5. Rigid body motion of the MalF Periplasmic region**

A large amplitude rigid body rotation of the second periplasmic region of MalF (MalF-P_2_ region) toward the membrane was observed in the **2ATP** state (see figure S5). As can be seen from figure S5, the MalF-P_2_ region rotation was detected for all ten replicates, and it takes 5 to 20 ns to be completed.


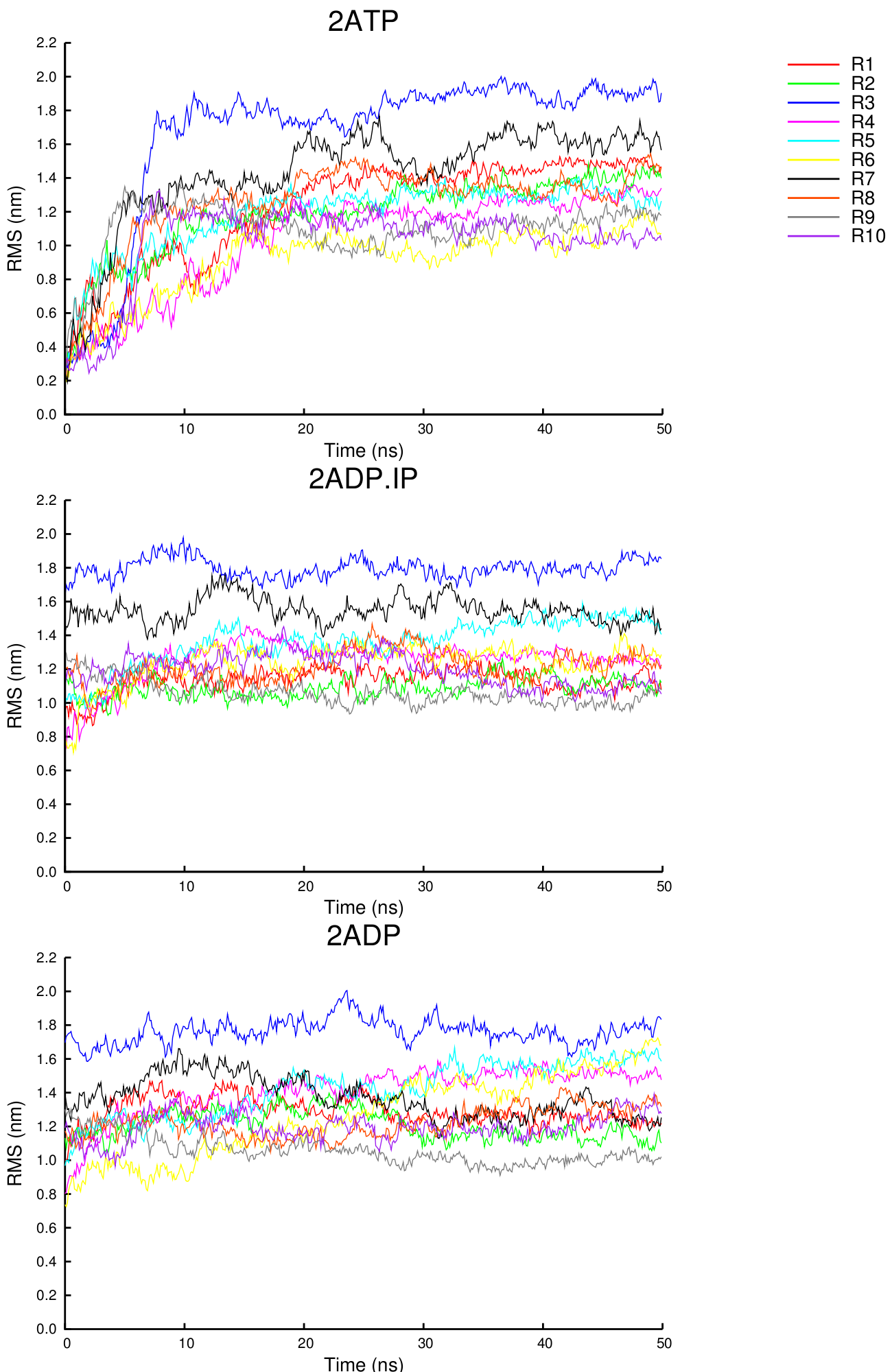


**Figure S5**- Time evolution of the RMSD for the C_α_ atoms of the MalF-P_2_ region with respect to its position in the crystal structure, after fitting to the Cα MalF core transmembrane region, for the **2ATP**, **2ADP.IP** and the **2ADP** states. Each point represents the average value over 100 ps.

Additionally, for the **2ADP.IP** and **2ADP** states, a high amplitude rigid body rotation (similar to the one reported for the **2ATP** state) was also observable for all replicates (see figures S5B and S5C).

**6. MalK dimer interface**

In order to determine if the MalK dimer dissociation was observable in any of the **2ATP**, **2ADP.IP** and **2ADP** replicates, the distance between the P-loop of one MalK monomer and the ABC signature motif of the other was determined. As can be seen in figure S6, no MalK dimer interface separation was detected in the **2ATP** and **2ADP.IP** states. In contrast, one of the ten **2ADP** replicates (replicate 8) clearly shows, after 37 ns of simulation, an interface opening in the nucleotide binding site 2 only.


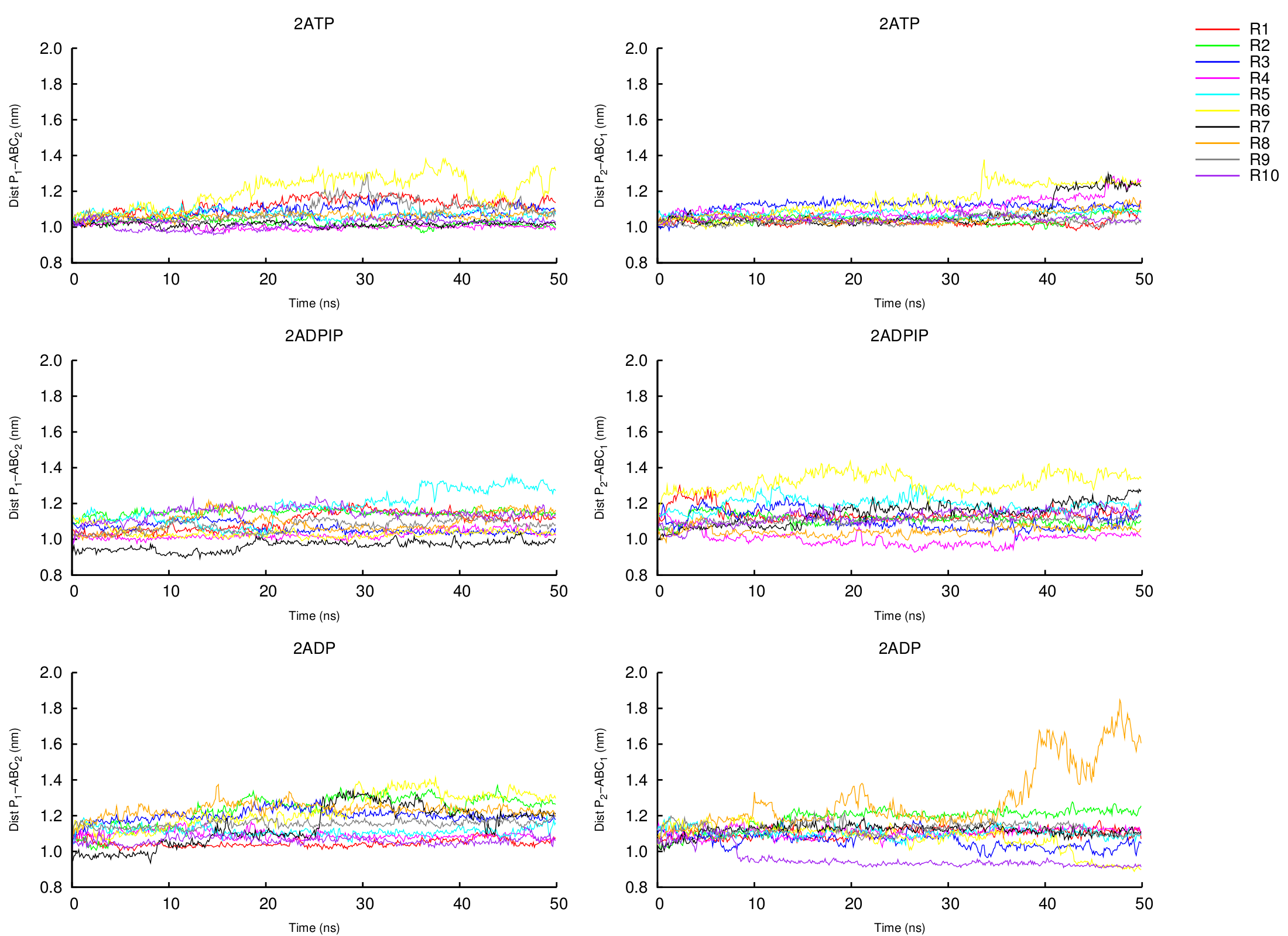


**Figure S6-** Distances, for all replicates, between the P-loop motif of one MalK monomer and the ABC-signature motif of the other. The graphics at the right side represent the distances for binding site 1, whereas the ones at the left represent the same measurement for binding site 2. Each point represents the average value over 100 ps.

The effect of the nucleotide binding site 2 dissociation in the MalF and MalG domains is small, and no significant conformational rearrangements in the transmembrane domains were detected, as can be confirmed in figure S7.


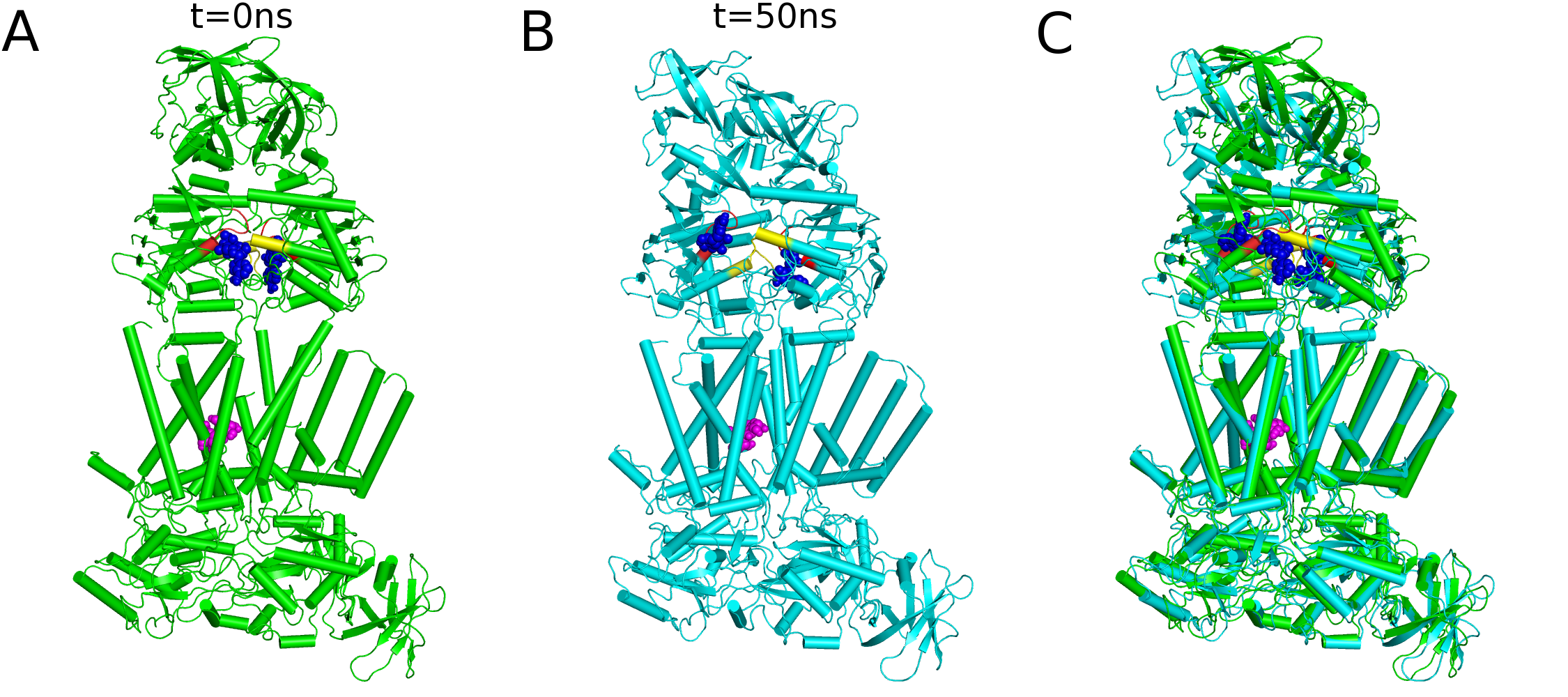


**Figure S7-** Opening of the MalK dimer interface and the absence of significant effects on the transmembrane domains. Snapshots of the **2ADP** replicate 8 simulation (the only replicate exhibiting a MalK dimer dissociation). The P-loop and the ABC motif are colored in red and yellow, respectively, whereas ADP and maltose are represented by dark blue and magenta spheres. **A**) Starting point (t=0ns). **B**) Final structure (after 50 ns). **C**) Superimposition of the starting and the final structures after fitting to the core transmembrane helices (residues 13-90 and 272-504 from MalF and 7-296 from MalG).

**7. Helical sub-domain rotation**

The helical sub-domain (HD) region has been identified in the last years as one of the regions presenting significant conformational changes during the ATP-cycle , and is most probably involved in the NBD-TMD communication mechanism . The rigid body outward rotation of the HD is thought to constitute one of the major structural differences between different nucleotide bound states in the NBDs of ABC transporters. In our simulations, in both post-hydrolysis states, a small amplitude rotation of the HD, relative to the pre-hydrolysis state, was observable (see figure S8).

**
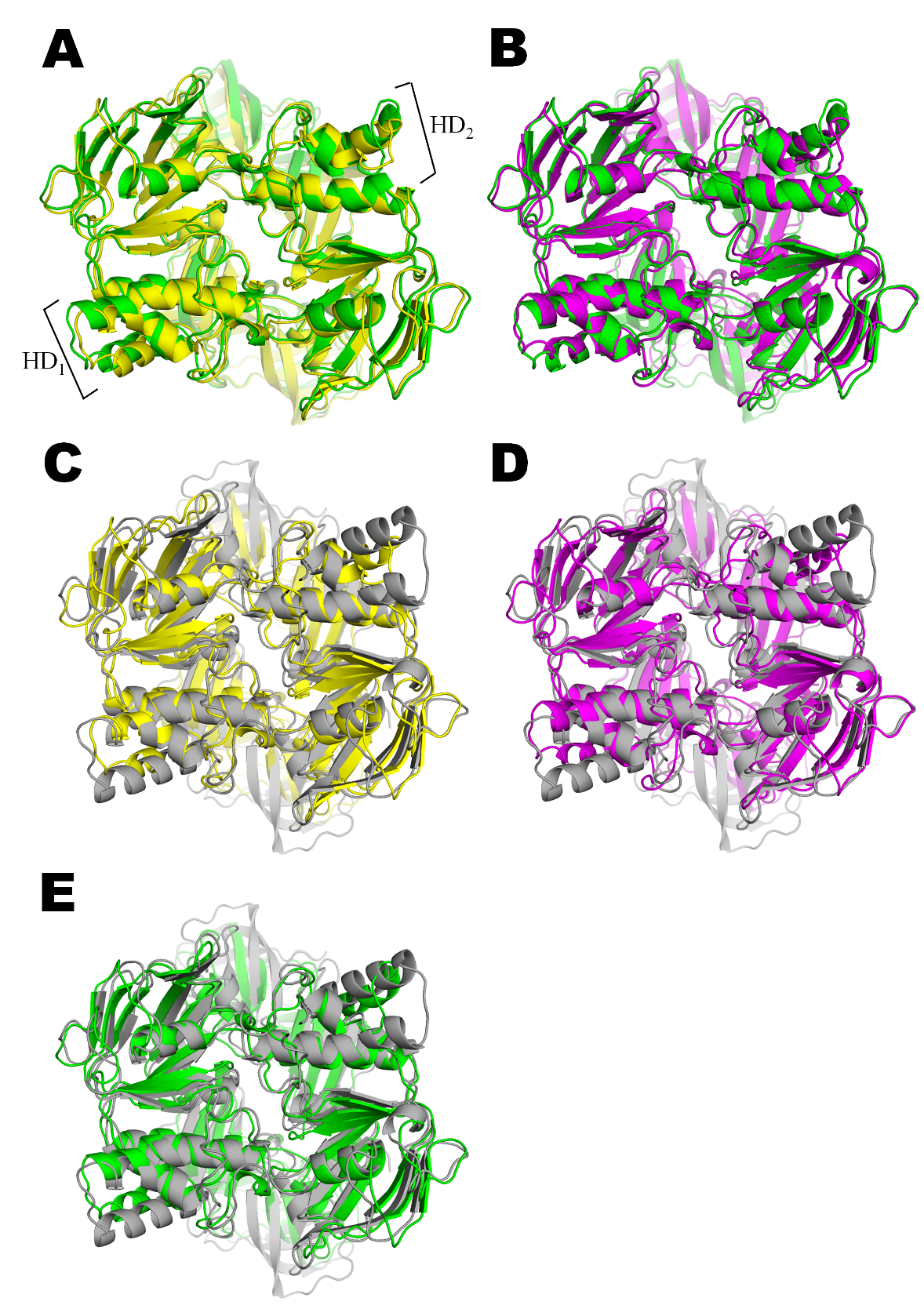
**

**Figure S8-** Helical sub-domain rotation upon hydrolysis and exit of the IP species. Figure **A, B-** Comparison between the average structures (obtained over the last 10 ns of simulation) of the **2ATP** (colored in green) and the: **A**- **2ADP.IP** state (colored in yellow). **B**- **2ADP** state (colored in magenta). Figures **C**, **D** and **E** correspond to the comparison between the X-ray structure (colored in gray) and the **2ADP.IP**, the **2ADP** and the **2ATP** states, respectively**.** All images were obtained after superimposition of the RecA-like sub-domains (residues 4 to 87 and 151 to 216) between the pre- and post-hydrolysis states. The regulatory domains are shown in the back with faded colors. For simplicity purposes, the MalF, MalG, MalE and the nucleotide species were not represented.

**8. The position of maltose during ATP hydrolysis**

In order to clarify if the ATP-hydrolytic cycle exerts any effect in the allocrite binding site, the maltose position was determined with respect to its initial coordinates for all replicates of all the states during the simulation time. As can be seen in figure S9, the maltose molecule does not abandon the allocrite binding site in the majority of the simulations (which corresponds to an RMSD lower than 0.5). However, two exceptions can be detected: one in the **2ATP** state (replicate 2) and the other in the **2ADP.IP** state (replicate 1). Moreover, a detailed view of the maltose movement from the allocrite binding site can be observed in figure S10.


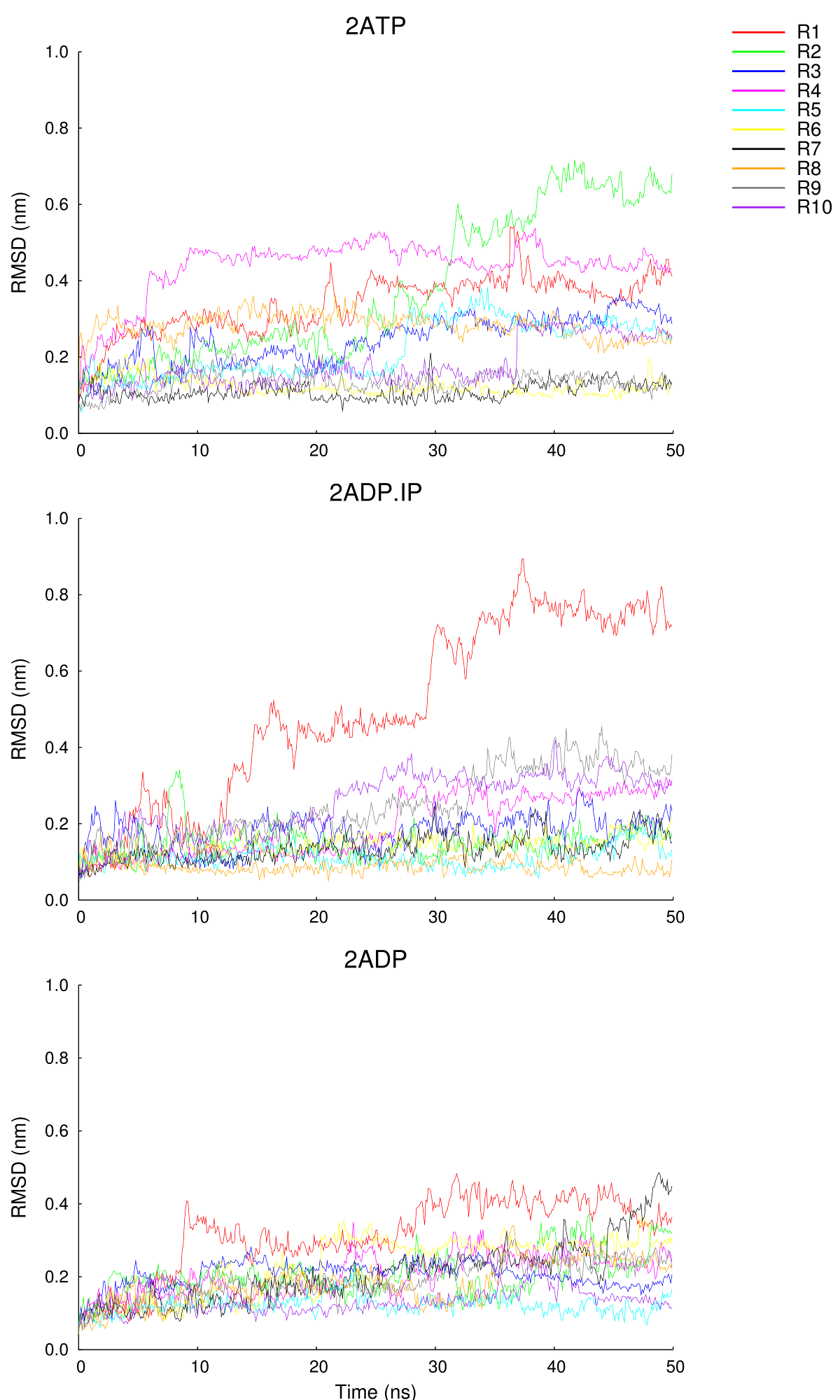


**Figure S9**- Time evolution of the position of maltose relative to its position in the starting structure, after root mean square fitting to the core transmembrane domains (MalF residue 13-90 and 272-504 and MalG residue 7-296) Cα atoms. Each point represents the average value over 100 ps


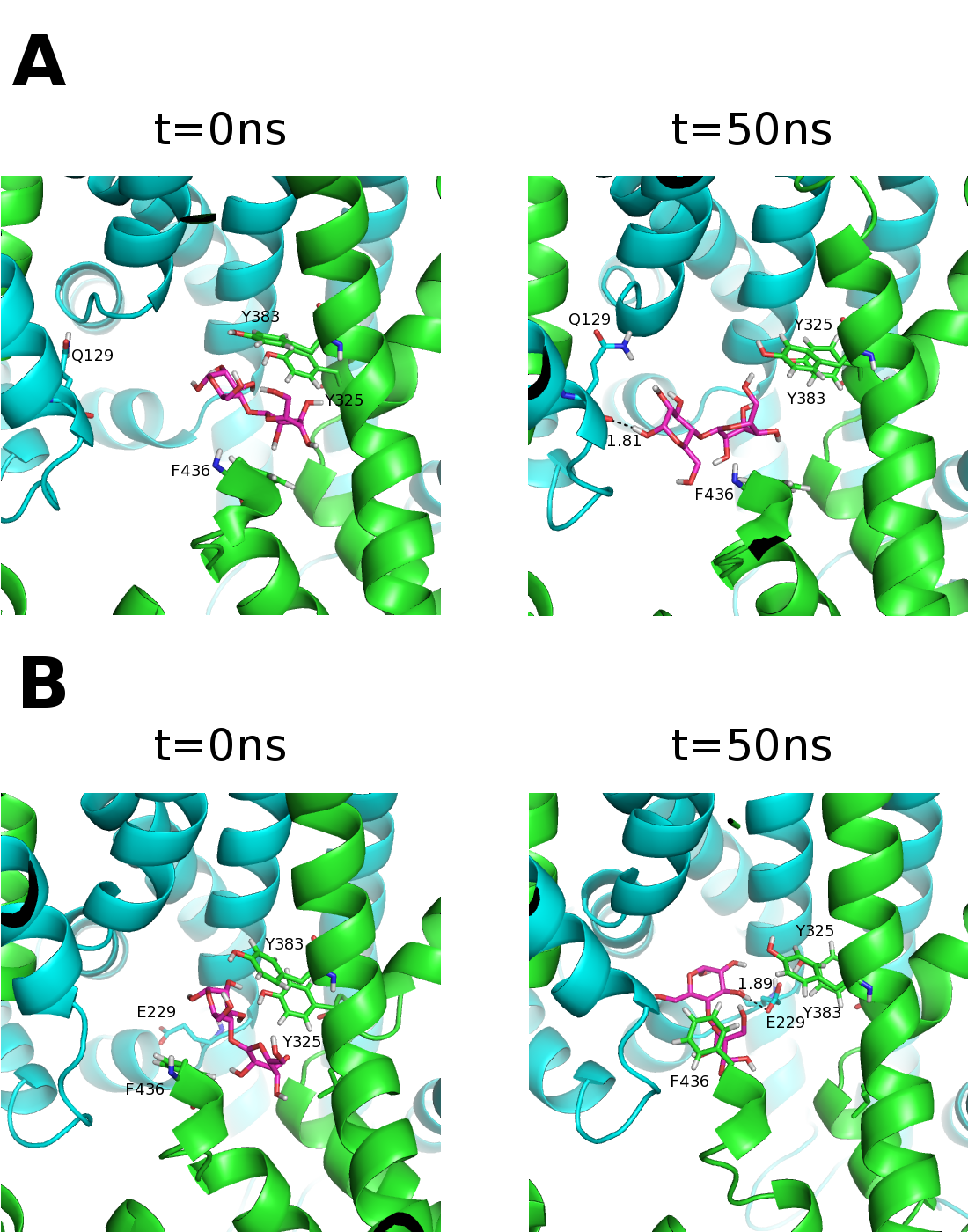


**Figure S10**- Snapshots of the two cases (replicate 2 of the **2ATP** state and replicate 1 of the **2ADP.IP** state) where maltose release from the allocrite binding site is observed. In these figures, MalF is colored in green, whereas MalG is colored in cyan. Some of the MalF (binding site) and MalG residues are represented in green and cyan sticks. The distance between maltose and the MalG residues is shown with a dashed line (Å). Snapshots of the allocrite binding site at the beginning (t=0 ns) and at the end (t=50 ns) of the simulation for the **A**) **2ATP** replicate 2. **B**) **2ADP.IP** replicate 1.

Additionally, in the replicates with a RMSD value lower than 0.2 nm, the maltose molecule does not move away from its starting position and the majority of the crystallographic interactions (like, the hydrogen bonds and the π-π interactions with the aromatic residues that form the binding site) are maintained. In the replicates presenting a RMSD between 0.2 and 0.5 nm, at least one of the π-π interactions with the aromatic residues (F436 or Y383) has disappeared, and maltose is able to rotate and to move slightly away from the binding site. Nevertheless, in all these replicates, maltose is still bound via hydrogen bonds and by this reason cannot freely diffuse away from the binding site.

The allocrite binding site region is not affected by ATP-hydrolysis or IP exit, since no significant differences in the binding site region were observed in the three studied states, at least within the simulated timescales (figure S11).


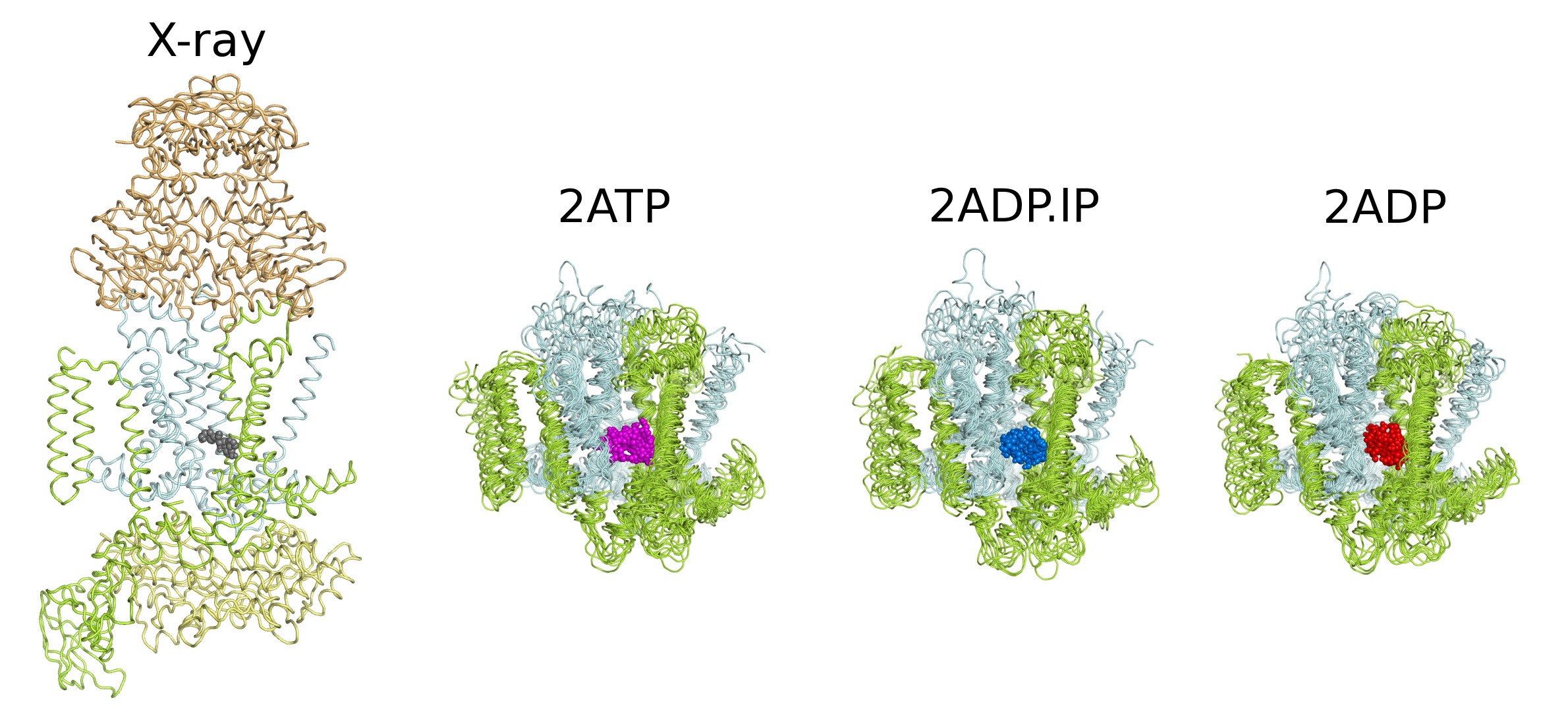


**Figure S11-** Maltose position in the transmembrane domains. In this figure, MalF is colored in green, whereas MalG is highlighted in blue. The left side image represents the X-ray structure with the MalK dimer, colored in light orange, and MalE colored in brown. The maltose molecule is represented with gray spheres. The remaining three images represent the last conformations (after 50 ns) for each one of the 10 replicates of the three simulated states, after fitting to the transmembrane core (MalF residues 13-90 and 272-504 and MalG 7-296). In the **2ATP, 2ADP.IP** and **2ADP,** only the MalF and MalG transmembrane regions are represented, for simplicity reasons, and the maltose molecule is shown in magenta, blue and red, respectively.

**9. Movie of the MalK dimer interface opening in the 2ADP state**

The MalK dimer interface dissociation was only clearly observable in one of replicate of the **2ADP** state (replicate 8). In this case, the binding site 2 opening is accompanied by the dissociation of ADP from the ABC signature motif, but not from the corresponding P-loop motif (which prevents ADP diffusing away from the binding site). For a clearer visualization of the atomic movements associated with the MalK dimer interface dissociation, we suggest the visualization of the following animation (Video S1)

**
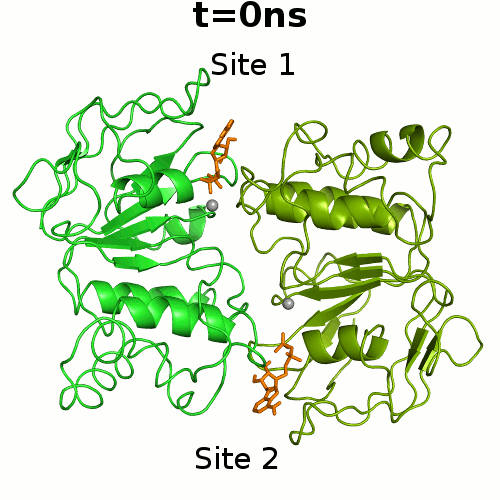
**

**Video S1-** This movie represents the MalK dimer interface opening for replicate 8 of the **2ADP** state. The MalK monomers are colored in green (the MalK_1_ is represented in light green, while the MalK_2_ in dark green), ADP is represented with orange sticks and Mg with gray spheres. For clarity purposes, the regulatory domains are not represented.

**REFERENCES**

1. Baptista AM, Soares CM (2001) Some theoretical and computational aspects of the inclusion of proton isomerism in the protonation equilibrium of proteins. J Phys Chem 105: 293-309.

2. Teixeira VH, Soares CM, Baptista AM (2002) Studies of the reduction and protonation behavior of tetraheme cytochromes using atomic detail. J Biol Inorg Chem 7: 200-216.

3. Bashford D, Gerwert K (1992) Electrostatic Calculations of the pKa Values of Ionizable Groups in Bacteriorhodopsin. J Mol Biol 224: 473-486.

4. Bashford D (1997) An Object-Oriented Programming Suite for Electrostatic Effects in Biological Molecules. In: Ishikawa Y, Oldehoeft RR, Reynders JVW, Tholburn M, editors. Scientific Computing in Object-Oriented Parallel Environments. Berlin: ISCOPE97, Springer. pp. 233-240.

5. Bashford D, Karplus M (1990) pKa's of ionizable groups in proteins: atomic detail from a continuum electrostatic model. Biochemistry 29: 10219-10225.

6. Van Der Spoel D, Lindahl E, Hess B, Groenhof G, Mark AE, et al. (2005) GROMACS: fast, flexible, and free. J Comput Chem 26: 1701-1718.

7. Berendsen H, Spoel D, Drunen R (1995) GROMACS 3.1.4. Comp Phys Comm 91: 43-56.

8. Lindahl E, Hess B, van der Spoel D (2001) GROMACS 3.0: a package for molecular simulation and trajectory analysis. J Mol Model 7: 306-317.

9. Berendsen HJC, Vanderspoel D, Vandrunen R (1995) Gromacs - a Message-Passing Parallel Molecular-Dynamics Implementation. Comput Phys Commun 91: 43-56.

10. van Gunsteren WF, Billeter SR, Eising AA, Hunenberger PH, Kruger P, et al. (1996) Biomolecular simulation: The GROMOS96 manual and user guide. Zurich, Groninger: BIOMOS b.v.

11. Scott WRP, Hünenberger PH, Tironi IG, Mark AE, Billeter SR, et al. (1999) The GROMOS biomolecular simulation program package. J Phys Chem 103: 3596-3607.

12. Oostenbrink C, Villa A, Mark AE, van Gunsteren WF (2004) A biomolecular force field based on the free enthalpy of hydration and solvation: the GROMOS force-field parameter sets 53A5 and 53A6. J Comput Chem 25: 1656-1676.

13. Oostenbrink C, Soares TA, van der Vegt NF, van Gunsteren WF (2005) Validation of the 53A6 GROMOS force field. Eur Biophys J 34: 273-284.

14. Chiu SW, Clark M, Balaji V, Subramaniam S, Scott HL, et al. (1995) Incorporation of surface tension into molecular dynamics simulation of an interface: a fluid phase lipid bilayer membrane. Biophys J 69: 1230-1245.

15. Koyama TM, Stevens CR, Borda EJ, Grobe KJ, Cleary DA (1999) Characterizing the Gel to Liquid Crystal Transition in Lipid-Bilayer Model Systems. Chem Educator 4: 12-15.

16. Nagle JF, Tristram-Nagle S (2000) Lipid bilayer structure. Curr Opin Struc Biol 10: 474-480.

17. Petrache HI, Dodd SW, Brown MF (2000) Area per lipid and acyl length distributions in fluid phosphatidylcholines determined by H-2 NMR spectroscopy. Biophys J 79: 3172-3192.

18. Wohlert J, Edholm O (2006) Dynamics in atomistic simulations of phospholipid membranes: Nuclear magnetic resonance relaxation rates and lateral diffusion. J Chem Phys 125: -.

19. Oldham ML, Khare D, Quiocho FA, Davidson AL, Chen J (2007) Crystal structure of a catalytic intermediate of the maltose transporter. Nature 450: 515-U517.

20. Campbell JD, Biggin PC, Baaden M, Sansom MS (2003) Extending the structure of an ABC transporter to atomic resolution: modeling and simulation studies of MsbA. Biochemistry 42: 3666-3673.

21. Haubertin DY, Madaoui H, Sanson A, Guerois R, Orlowski S (2006) Molecular dynamics simulations of E. coli MsbA transmembrane domain: formation of a semipore structure. Biophys J 91: 2517-2531.

22. Aittoniemi J, de Wet H, Ashcroft FM, Sansom MS (2010) Asymmetric switching in a homodimeric ABC transporter: a simulation study. PLoS Comput Biol 6: e1000762.

23. Kabsch W, Sander C (1983) Dictionary of protein secondary structure: pattern recognition of hydrogen-bonded and geometrical features. Biopolymers 22: 2577-2637.

24. Yuan YR, Blecker S, Martsinkevich O, Millen L, Thomas PJ, et al. (2001) The crystal structure of the MJ0796 ATP-binding cassette. Implications for the structural consequences of ATP hydrolysis in the active site of an ABC transporter. J Biol Chem 276: 32313-32321.

25. Karpowich N, Martsinkevich O, Millen L, Yuan YR, Dai PL, et al. (2001) Crystal structures of the MJ1267 ATP binding cassette reveal an induced-fit effect at the ATPase active site of an ABC transporter. Structure (Camb) 9: 571-586.

26. Smith PC, Karpowich N, Millen L, Moody JE, Rosen J, et al. (2002) ATP binding to the motor domain from an ABC transporter drives formation of a nucleotide sandwich dimer. Mol Cell 10: 139-149.

27. Chen J, Lu G, Lin J, Davidson AL, Quiocho FA (2003) A tweezers-like motion of the ATP-binding cassette dimer in an ABC transport cycle. Mol Cell 12: 651-661.

28. Lu G, Westbrooks JM, Davidson AL, Chen J (2005) ATP hydrolysis is required to reset the ATP-binding cassette dimer into the resting-state conformation. Proc Natl Acad Sci U S A 102: 17969-17974.

29. Jones PM, George AM (2007) Nucleotide-dependent allostery within the ABC transporter ATP-binding cassette: a computational study of the MJ0796 dimer. J Biol Chem 282: 22793-22803.

30. Oliveira AS, Baptista AM, Soares CM (2010) Insights into the molecular mechanism of an ABC transporter: conformational changes in the NBD dimer of MJ0796. J Phys Chem B 114: 5486-5496.

31. Hyde SC, Emsley P, Hartshorn MJ, Mimmack MM, Gileadi U, et al. (1990) Structural model of ATP-binding proteins associated with cystic fibrosis, multidrug resistance and bacterial transport. Nature 346: 362-365.
